# Oncolytic HSV-1 expressing FLT3L kills melanoma, glioblastoma, and pancreatic cancer cells *in vitro* and induces immunogenic cell death

**DOI:** 10.1016/j.omton.2025.201031

**Published:** 2025-08-09

**Authors:** Sandra Tuyaerts, Xenia Geeraerts, Alberto Reale, Latoya Stevens, Giada Bertazzon, Jack Brons, Toon Janssen, Ivan Van Riet, Arianna Calistri, Bart Neyns

**Affiliations:** 1 Laboratory for Medical and Molecular Oncology (LMMO), Translational Oncology Research Center (TORC), Vrije Universiteit Brussel (VUB), Brussels, Belgium; 2 Department of Medical Oncology, Universitair Ziekenhuis Brussel (UZ Brussel), Brussels, Belgium; 3 Department of Molecular Medicine, University of Padua, 35121 Padua, Italy; 4 Laboratory for Molecular and Cellular Therapies (LMCT), Translational Oncology Research Center (TORC), Vrije Universiteit Brussel (VUB), Brussels, Belgium; 5 Department of Genetics, Universitair Ziekenhuis Brussel (UZ Brussel), Vrije Universiteit Brussel (VUB), Brussels, Belgium; 6 Department of Hematology and Stem Cell Laboratory, Universitair Ziekenhuis Brussel (UZ Brussel), Brussels, Belgium

**Keywords:** MT: Regular Issue, oncolytic virus, herpes simplex virus type 1, FLT3L, conventional dendritic cells, immunogenic cell death, melanoma, glioblastoma, pancreatic carcinoma

## Abstract

Oncolytic viruses (OVs) are promising anti-cancer agents designed to induce cancer cell death while simultaneously stimulating immune responses through encoded transgenes. FMS-like tyrosine kinase 3 ligand (FLT3L), a critical cytokine in dendritic cell (DC) biology, was incorporated into a genetically engineered OV derived from herpes simplex virus type 1. This virus was modified with deletions in the *γ34.5* neurovirulence gene and the *US12* gene. Treatment with the FLT3L-encoding OV resulted in a time- and dose-dependent increase in FLT3L secretion and inhibition of cell growth across all tested cell lines, although sensitivity varied among the lines. Susceptibility to oncolysis correlated with the expression levels of *NECTIN1*, *NECTIN2*, and *ITGB6*. OV-induced lysates from melanoma and ASPC1 cell lines showed minimal effects on the phenotype of conventional DCs (cDCs). However, oncolysates significantly increased the secretion of interferon (IFN)-λ1 and IFN-α-2a, particularly using BXPC3 oncolysates. Additionally, treatment of pancreatic cancer and 938-mel cell lines with the FLT3L-expressing OV elevated ATP levels but did not affect HMGB1 release. In conclusion, this study demonstrates the dual oncolytic and immunogenic potential of an FLT3L-encoding OV, particularly on cDCs. These findings support the further development of this approach as a novel cancer immunotherapy.

## Graphical abstract

**Figure:**
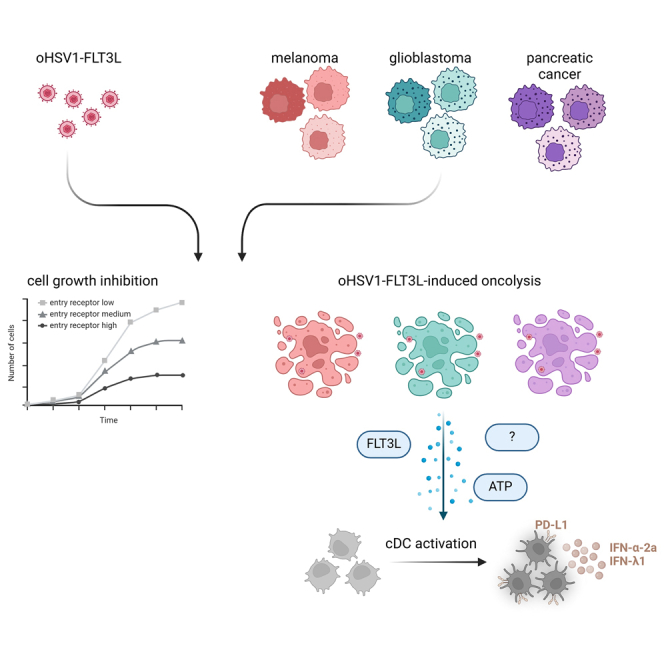

## Teaser

Tuyaerts and colleagues developed an FLT3L-encoding herpes simplex virus type 1, capable of inhibiting growth of melanoma, glioblastoma, and pancreatic adenocarcinoma cell lines. The oncolytic effect correlated with viral entry receptor expression by the cancer cell lines. The induced cell death was immunogenic, leading to partial activation of dendritic cells.

## Introduction

Oncolytic virotherapy is under development to induce local tumor destruction through preferential infection and killing of tumor cells.^1^ Oncolytic viruses (OVs) also induce immunogenic cell death (ICD) by releasing pathogen-associated molecular patterns (PAMPs) and damage-associated molecular patterns (DAMPs), which elicit an anti-tumor immune response against tumor-associated antigens [including (*neo*)antigenic epitopes] from the dying cancer cells.^1^ An additional appealing feature of OVs is the capacity to encode therapeutic transgenes into the viral genome.^2^ A prototype OV is talimogene laherparepvec (T-VEC), the first US Food and Drug Administration - and European Medicine Agency-approved OV, which is a herpes simplex virus 1 (HSV1) genetically modified to reduce its pathogenicity and to encode granulocyte macrophage colony stimulating factor (GM-CSF). Incorporation of GM-CSF has shown to enhance local accumulation of immune cells into the tumor microenvironment (TME),^1^^,^^3^^,^^4^^,^^5^ including dendritic cells (DCs).

DCs are central players in mediating immune cell influx into tumors. As conceptualized in the cancer-immunity cycle,^6^^,^^7^ conventional DCs (cDCs) are key cellular components that initiate and intratumorally relicense anti-tumor T cells yielding effective tumor eradication.^8^^,^^9^^,^^10^^,^^11^ The presence of DCs in the TME correlates with T cell infiltration into tumors. In particular, cDCs, including the BDCA-3 (CD141)^+^ cDC1 and BDCA-1 (CD1c)^+^ cDC2 subsets, are key to tumor antigen cross-presentation. The absence of cDCs in the TME has demonstrated to correlate with a worse outcome.^12^^,^^13^ Moreover, evidence suggests that anti-programmed cell death (PD)-1 efficacy depends on a DC-T-cell licensing loop driven by interleukin (IL)-12 and interferon (IFN)-γ, confirming a central role of DCs in the activity of ICB.^14^ Moreover, in animal models it has been reported that exclusion of cDCs from the TME was detrimental to ICB activity due to defective activation of cytotoxic T lymphocytes, allowing metastases to escape anti-tumor immune responses.^15^

Our group previously performed a clinical trial (myDCTV) investigating the combined intratumoral administration of BDCA-1 (CD1c)^+^ cDCs or combined BDCA-1 (CD1c)^+^, and BDCA-3 (CD141)^+^ cDCs with T-VEC.^16^ Tumor regression was observed in 4 of 12 metastatic melanoma patients, including 2 patients who showed a complete response that is still ongoing more than 5 years after the initiation of study treatment. Furthermore, on-treatment biopsies revealed a strong infiltration by inflammatory cells in regressing lesions.^16^ We also reported preclinical data showing that T-VEC induced ICD in human melanoma cells, leading to partial maturation of DCs and uptake and cross-presentation of melanoma-derived tumor antigens to tumor-specific CD8^+^ T cells.^17^ Non-responding patients from the myDCTV trial showed a non-T cell-inflamed TME with few lymphocytes and absence of PD ligand 1 (PD-L1) expression or developed a downregulation of the antigen-presenting machinery apparatus.^16^

Although the OPTiM phase 3 trial showed that T-VEC does improve long-term efficacy when compared with GM-CSF administration alone,^18^ in contrast a phase 3 study comparing pembrolizumab with and without T-VEC in unresectable stage IIB-IVM1 melanoma did not show a significant improvement of progression-free survival or overall survival.^19^ In addition, a critical re-evaluation of the most recent research on GM-CSF has cast doubt on its usefulness in cancer immunotherapy, considering that this factor also has pro-tumorigenic effects.^20^

Human FMS-like tyrosine kinase 3 ligand (FLT3L) emerged as an intriguing cytokine given its various functions, particularly relating to DCs. Prior studies have demonstrated its impact on the development, maintenance, and differentiation of DC progenitors into DC subsets such as cDCs.^21^ Additionally, FLT3L influences the activation and expansion of DC populations in mouse melanoma models,^15^ as well as at tumor sites.^22^ Therefore, the FLT3L signaling cascade is an interesting target to increase the number of cross-presenting DCs that, in turn, could lead to an improved anti-tumor immune response.^23^ Furthermore, considering that FLT3L expression is preserved in terminally differentiated DCs, it might have a functional impact on differentiated DCs subsets as well.^22^^,^^24^ We hypothesize that, following the initial intratumoral administration of cDCs in combination with an FLT3L-encoding OV, a continuous influx of cDCs into the tumor could be driven by FLT3L released from infected/killed tumor cells. In turn, these DCs are anticipated to reinvigorate T cells to tumor antigens released by the infected/killed tumor cells.

In this study, we report on the design of a new HSV1 construct, incorporating FLT3L instead of GM-CSF. This oHSV1-FLT3L has the same backbone as T-VEC, with double deletion of *γ34.5* and *US12* and the insertion of *FLT3LG* in the *UL55–UL56* intergenic region. Deletion of the *γ34.5* neurovirulence gene blocks both the protein kinase R (PKR) pathway activated by double-stranded DNA (dsRNA) and autophagy, while deletion of *US12* prevents antigen presentation by infected cells. The deletion of *US12* alters the kinetics of another viral gene, *US11*, allowing it to interfere with the cellular PKR pathway.^25^ We assessed its oncolytic effect on melanoma, glioblastoma, and pancreatic carcinoma cell lines and studied the interaction between oHSV1-FLT3L-induced oncolysates and human cDC subsets.

## Results

### Treatment with oHSV1-FLT3L inhibits tumor cell growth and induces FLT3L production

The oncolytic activity of oHSV1-FLT3L on melanoma, glioblastoma, and pancreatic cancer cell lines was determined by evaluating its inhibitory effect on cancer cell growth using Incucyte live cell imaging analysis. Treatment with different multiplicities of infection (MOIs) (0.001, 0.01, 0.1, 1, and 10) induced a dose- and time-dependent effect on cell confluence for all tested cell lines, except for MiaPaca2 where the effect on confluence was limited (Figure S1).

**Figure 1:**
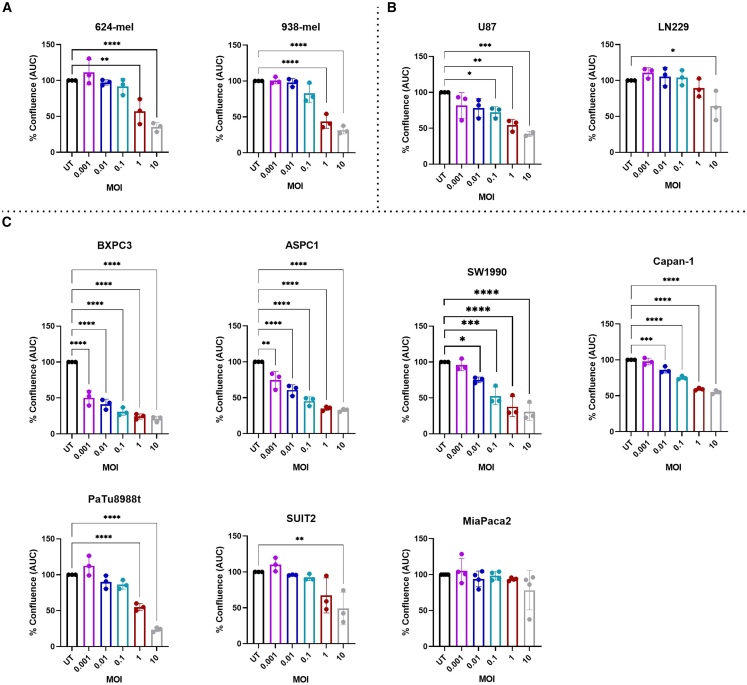
Inhibition of cell growth induced by oHSV1-FLT3L in different cancer cell lines The percent confluence is displayed as the AUC normalized to the untreated (UT) condition for (A) melanoma cell lines (624-mel and 938-mel), (B) glioblastoma cell lines (U87 and LN229), (C) pancreatic ductal adenocarcinoma cell lines (BXPC3, ASPC1, Capan-1, SW1990, PaTu8988t, SUIT2, and MiaPaca2). Bars depict the mean ± SD of three biological repeats with each individual data point depicting the mean of three technical repeats, except for U87 MOI 10 with two and MiaPaca2 with four repeats. An ordinary one-way ANOVA with Dunnett’s multiple comparisons test was performed to compare the UT condition with every MOI. ∗*p* ≤ 0.05, ∗∗*p* ≤ 0.01, ∗∗∗*p* ≤ 0.001, ∗∗∗∗*p* ≤ 0.0001.

Subsequently, the area under the curve (AUC) of the confluence was calculated for each MOI and normalized to the AUC of the untreated condition to quantify the OV-induced effect. Treatment with oHSV1-FLT3L led to a significant decrease of confluence in both melanoma cell lines (624-mel and 938-mel) compared with the untreated condition, with significant inhibition observed as from MOI 1 (Figure 1A). Glioblastoma cell lines exhibited variable responses, with U87 displaying a significant decrease in cell growth from MOI 0.1 onward, while the LN229 cell line was only susceptible to infection at the highest MOI (Figure 1B). Pancreatic cancer cell lines also showed heterogeneous patterns of susceptibility to OV-mediated inhibition, with BXPC3 and ASPC1 showing significant cell growth inhibition at an MOI of 0.001, and Capan-1 and SW1990 cells exhibiting inhibition starting from an MOI of 0.01. The PaTu8988T cell line displayed a significant decrease starting from an MOI of 1, while SUIT2 was only susceptible to OV treatment at the highest MOI. Notably, MiaPaca2 was the least susceptible to oHSV1-FLT3L treatment; none of the tested MOIs induced a significant decrease in cell growth (Figure 1C). For most cell lines, the growth inhibitory effect induced by oHSV1-FLT3L was comparable with T-VEC, but for 624-mel, 938-mel, Capan-1, PaTu8988t, and MiaPaca2, T-VEC induced a stronger growth inhibition at one or more MOIs (Figure S2).

To confirm that OV-mediated inhibition of cell growth ultimately resulted in the induction of cell death, cell viability was analyzed at different MOIs (0.001, 0.01, 0.1, 1, and 10), by using the cytotox dye in Incucyte live cell imaging analysis. For most cell lines (except MiaPaca-2), we observed an MOI-dependent increase in cell death upon treatment with either oHSV1-FLT3L or T-VEC (Figure S3). Cell death occurred at later time points (>50 h) compared with growth inhibition, except for 938-mel, BXPC3, and ASPC1, where cell death increased before 50 h for the highest MOIs. Upon calculation of the AUC of the normalized cell death, we indeed observed an MOI-dependent increase in cell death, which was significant for LN229, ASPC1, and Capan-1 (Figure S4).

**Figure 2:**
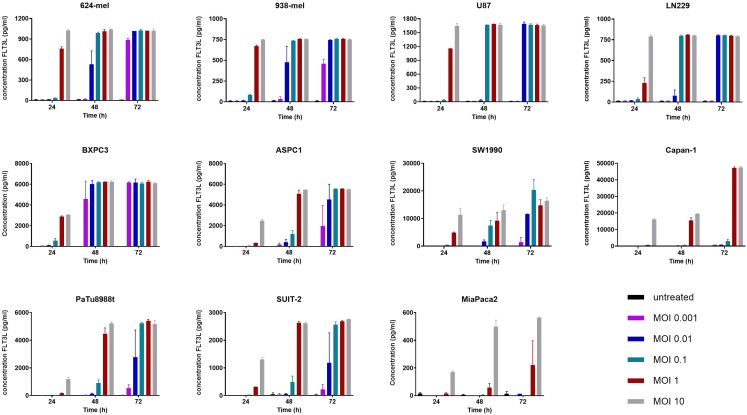
Induction of FLT3L secretion by oHSV1-FLT3L in different cancer cell lines Cancer cell lines were treated at different MOIs (0.001–10) and supernatant was harvested after 24, 48, and 72 h. FLT3L was detected in the supernatant of oHSV1-FLT3L-treated tumor cell lines by ELISA. Graphs depict one representative experiment out of two or three biological repeats with bars representing the mean ± SD of three technical replicates. Statistical analysis was performed using a mixed-effects model to assess the effects of time and MOI. Statistical analysis and *p* values are shown in Tables 1, S1, and S2.

To assess transgene expression as a marker of productive infection by the oHSV1-FLT3L virus in cancer cells, FLT3L secretion in the supernatant was measured using ELISA. Figure 2 illustrates FLT3L secretion from various cancer cell lines infected with oHSV1-FLT3L at different MOIs across multiple time points. The FLT3L secretion profile for most cell lines closely followed their growth inhibition patterns after oHSV1-FLT3L infection, with the exception of MiaPaca2, where a dose-dependent effect on FLT3L secretion was observed in two of three experiments (Figure S5). For all cell lines, FLT3L secretion exhibited a significant time- and MOI-dependent effect (Tables 1, S1, and S2). Additionally, a statistically significant interaction between time and MOI effects on FLT3L secretion was observed (Table 1).

**Table 1:** Mixed-effects analysis of FLT3L ELISA

	Fixed effects (type III)	*p* Value	*p* value summary	F (DFn, DFd)
624-mel	time	<0.0001	∗∗∗∗	F (2, 18) = 374.9
	MOI	<0.0001	∗∗∗∗	F (5, 12) = 406.2
	time × MOI	<0.0001	∗∗∗∗	F (10, 18) = 82.44
938-mel	time	<0.0001	∗∗∗∗	F (2, 30) = 161.9
	MOI	<0.0001	∗∗∗∗	F (5, 30) = 269.5
	time × MOI	<0.0001	∗∗∗∗	F (10, 30) = 39.08
LN229	time	<0.0001	∗∗∗∗	F (2, 22) = 1,436
	MOI	<0.0001	∗∗∗∗	F (5, 12) = 2,085
	time × MOI	<0.0001	∗∗∗∗	F (10, 22) = 410.7
U87	time	<0.0001	∗∗∗∗	F (2, 18) = 4,407
	MOI	<0.0001	∗∗∗∗	F (5, 12) = 7,797
	time × MOI	<0.0001	∗∗∗∗	F (10, 18) = 1,593
BXPC3	time	<0.0001	∗∗∗∗	F (2, 35) = 463.1
	MOI	<0.0001	∗∗∗∗	F (5, 35) = 155.6
	time × MOI	<0.0001	∗∗∗∗	F (10, 35) = 24.58
ASPC1	time	<0.0001	∗∗∗∗	F (2, 20) = 113.6
	MOI	<0.0001	∗∗∗∗	F (5, 12) = 49.85
	time × MOI	<0.0001	∗∗∗∗	F (10, 20) = 13.38
SUIT2	time	<0.0001	∗∗∗∗	F (2, 18) = 63.58
	MOI	<0.0001	∗∗∗∗	F (5, 12) = 73.29
	time × MOI	<0.0001	∗∗∗∗	F (10, 18) = 12.40
Capan-1	time	<0.0001	∗∗∗∗	F (2, 24) = 3,851
	MOI	<0.0001	∗∗∗∗	F (5, 12) = 3,952
	time × MOI	<0.0001	∗∗∗∗	F (10, 24) = 1,386
PaTu8988t	time	<0.0001	∗∗∗∗	F (2, 35) = 154.9
	MOI	<0.0001	∗∗∗∗	F (5, 35) = 93.91
	time × MOI	<0.0001	∗∗∗∗	F (10, 35) = 24.50
SW1990	time	<0.0001	∗∗∗∗	F (2, 36) = 119.5
	MOI	<0.0001	∗∗∗∗	F (5, 36) = 107.1
	time × MOI	<0.0001	∗∗∗∗	F (10, 36) = 17.23
MiaPaca2	time	0.0087	∗∗	F (2, 9) = 8.430
	MOI	<0.0001	∗∗∗∗	F (5, 11) = 49.02
	time × MOI	0.0044	∗∗	F (10, 9) = 6.661

In conclusion, we observed a time- and MOI-dependent effect from oHSV1-FLT3L on cancer cell growth and FLT3L production that was variable among different cell lines.

### Genetic characteristics of cancer cells that predict susceptibility to oHSV1-FLT3L infection

Given the great variability in the susceptibility of different cancer cell lines to oHSV1-FLT3L-mediated growth inhibition, we sought to determine whether factors such as the expression of viral entry receptors, antiviral machinery genes, or mutational status influenced their sensitivity. We performed bulk RNA sequencing on uninfected cell lines and analyzed the gene expression of selected viral entry receptors and antiviral machinery genes. For this analysis, the cell lines were categorized from high to low susceptibility to oHSV1-FLT3L-mediated growth inhibition based on the inhibition of cell growth in previous Incucyte imaging experiments: BXPC3 > ASPC1 > SW1990 > Capan-1 > U87 > 938-mel > PaTu8988t > 624-mel > SUIT2 > LN229 > MiaPaca-2 (Table S3), and the correlation between gene expression and growth inhibition from Inucyte imaging was calculated.

**Figure 3:**
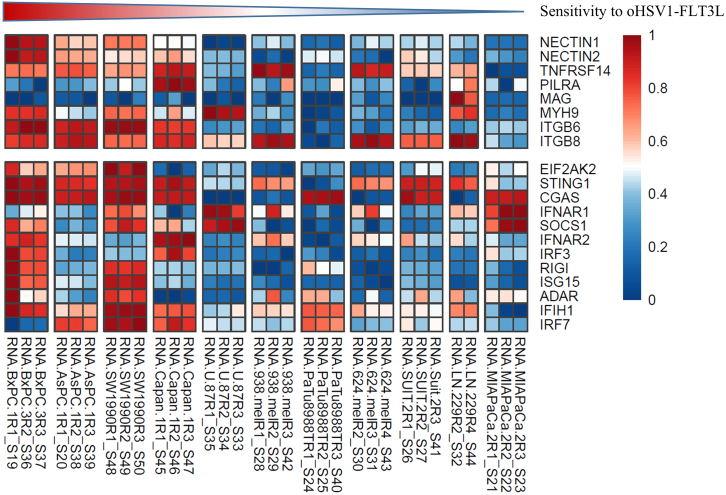
Expression profile of viral entry receptors and antiviral machinery genes by different cancer cell lines in relation to their sensitivity to infection with oHSV1-FLT3L Raw read counts were normalized using DESeq2 and log-transformed. Normalized gene expression levels were scaled and plotted in a heatmap. Data are shown as biological replicates per cell line (*n* = 3 per cell line, except for LN229 where *n* = 2 due to a technical error) and cell lines are ordered from high to low sensitivity to infection with oHSV1-FLT3L.

Entry of HSV into cells is mediated by the recognition/binding of viral glycoproteins to different receptors expressed by host cells. Expression of viral entry receptors among the different cell lines was assessed by bulk RNA sequencing and was very heterogeneous (Figure 3, top). Both melanoma cell lines showed a comparable viral entry receptor expression profile, with high expression of *TNFRSF14* and *ITGB8* and low expression of all other genes, which might explain their similar sensitivity to infection with oHSV1-FLT3L. On the other hand, both glioblastoma cell lines showed a more differential expression profile with high expression of *MYH9* and *ITGB8* in U87 cells, while LN229 cells showed higher levels of *TNFRSF14*, *PILRA*, *MAG*, *MYH9*, and *ITGB8*. Finally, the pancreatic cancer cell lines were also highly heterogeneous for viral entry receptor expression. The four most susceptible cell lines showed high expression of all entry receptors except *PILRA* and *MAG* (BXPC3, ASPC1, SW1990) or *MAG* (Capan-1). The cell lines Patu8988t and MiaPaca-2 showed low expression of all viral entry receptors, while SUIT2 showed high *ITGB8* expression and moderate expression of *NECTIN2* and *TNFRSF14*. Spearman correlation analysis revealed a strong negative correlation between the rank-based AUC of tumor cell growth (as shown in Table S3) and the expression of *NECTIN2* (r = −0.7182; *p* = 0.0162) and *ITGB6* (r = −0.6; *p* = 0.0562), as well as a moderate negative correlation with *NECTIN1* (r = −0.6545; *p* = 0.0336). These findings suggest that the expression of these markers may serve as a potential predictor of susceptibility to oHSV1-FLT3L infection, which would, however, need to be validated in additional experiments with cell lines wherein these genes are knocked out or down.

The preferential replication of OVs in tumor cells is thought to occur in part because of deficiencies in antiviral machinery in tumor cells. HSV-1 has been described to be detected in host cells by two major antiviral pathways, the PKR (*EIF2AK2*) and cGAS/STING pathways. The expression levels of genes in these pathways in the various cell lines was determined by bulk RNA sequencing and compared with the oHSV1-FLT3L-mediated inhibition of cell growth (Figure 3, bottom). Both melanoma cell lines showed again a very comparable expression pattern of antiviral genes, with low expression of most genes, except for *STING1*, *IFNAR1*, *IFNAR2*, and *IFIH1*, which were moderately expressed. The glioblastoma cell lines showed a more diverse expression profile, with U87 showing high expression of *IFNAR1* and *SOCS1* and low expression of all other antiviral genes, while LN229 showed high expression of *STING1* and *IFIH1* and moderate expression of *IFNAR1*. The pancreatic cancer cell lines could broadly be divided into two groups: one group expressing high levels of several antiviral genes (BXPC3, ASPC1, SW1990, and Capan-1) and another group expressing only a few antiviral genes (PaTu8988t, SUIT2, and MiaPaca-2). Surprisingly, the pancreatic cell lines with the highest expression levels of antiviral genes were most sensitive to oHSV1-FLT3L infection. Spearman correlation analysis showed a strong negative correlation between the effect of oHSV1-FLT3L on the rank-based AUC of the tumor cell growth (shown in Table S3) and expression of *RIG-I* (r = −0.6727; *p* = 0.0277), *ISG15* (r = −0.6909; *p* = 0.0226), and *OAS1* (r = −0.6636; *p* = 0.0306; data not shown).

From public databases, we collected the mutational status of the distinct cancer cell lines, except for 938-mel, for which no data were available (Table S4). Most cell lines harbored a *TP53* abnormality, except for U87. The 624-mel cell line contained a mutated *BRAF* gene (Val600Glu), while both glioblastoma cell lines are characterized by a *TERT* promotor mutation and a *CDKN2C* deletion. All pancreatic cancer cell lines, except BXPC3, are characterized by a *KRAS* mutation. We could not identify a specific genetic abnormality that correlated with sensitivity to oHSV1-FLT3L infection (Table S4).

### Conventional BDCA-1^+^/BDCA-3^+^ cDCs are susceptible to oHSV1-FLT3L infection

**Figure 4:**
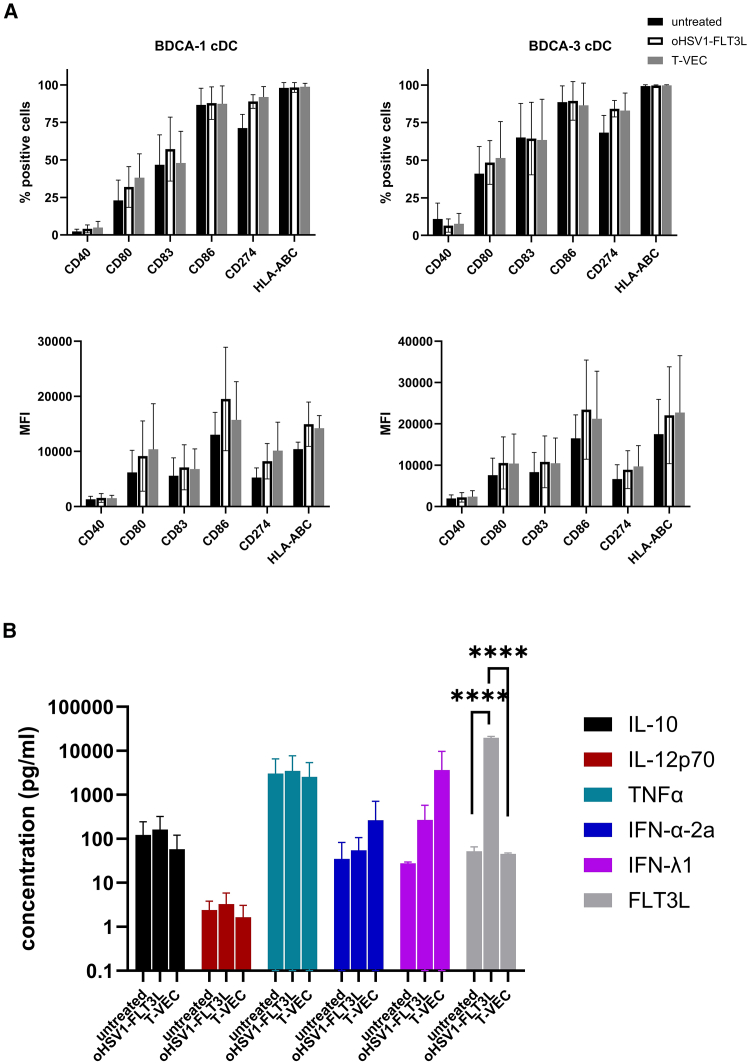
Effect of oHSV1-FLT3L on BDCA-1^+^/BDCA-3^+^ cDC phenotype and cytokine secretion BDCA-1^+^/BDCA3^+^ cDCs were cultured with or without oHSV1-FLT3L or T-VEC (MOI 1) for 24 h. (A) The phenotype of BDCA-1^+^/BDCA3^+^ cDCs was assessed upon analysis of DC maturation markers (CD274, CD86, CD83, CD40, CD80, and HLA-ABC) via flow cytometry. The percentage of positive cells (top) and MFI (bottom) of every marker is displayed for BDCA-3^+^ cDC (right) and BDCA-1^+^ cDC (left). Bars represent the mean ± SD of five independent experiments. Statistical analysis was performed using an ordinary two-way ANOVA with Sidák’s multiple comparison test. MFI, mean fluorescence intensity. (B) Cytokine secretion in the supernatants from BDCA-1^+^/BDCA3^+^ cDCs cultured with or without oHSV1-FLT3L or T-VEC was analyzed using a custom MesoScale Diagnostics U-plex assay to detect IL-10, IL-12p70, TNF-α, IFN-α-2a, IFN-λ1, and FLT3L. The graphs represent the mean ± SD from four independent experiments and each sample was tested in duplicate. For some conditions the values were undetectable as the concentration was below the fit curve, in which case we substituted this by the low detection limit/sqrt (2). If the values were above the fit curve, the highest detection limit was used. Statistical analysis was performed using an ordinary two-way ANOVA with Sidák’s multiple comparison test. ∗∗∗∗*p* ≤ 0.0001

Since cDCs play an essential role in enhancing anti-tumor immune responses, we assessed the effect of oHSV1-FLT3L on their phenotype, cytokine secretion, and viability. Culturing purified BDCA-1^+^/BDCA-3^+^ cDCs for 24 h in medium alone already induced upregulation of certain maturation markers (CD80, CD83, CD86, and CD274), which only increased further for CD80 using R848/Poly(I:C) as a positive control (Figures S6A–S6D). However, we noted an increased secretion of IL-10, IL-12p70, tumor necrosis factor (TNF)-α, IFN-α-2a, and IFN-λ1 when BDCA-1^+^/BDCA-3^+^ cDCs were cultured in the presence of R848/Poly(I:C) compared with medium alone (Figure S6E), indicating that functional maturation was only induced upon addition of the maturation stimulus R848/Poly(I:C). Upon culture of BDCA-1^+^/BDCA-3^+^ cDCs in the presence of oHSV1-FLT3L at an MOI of 1, we did not observe significant changes in the phenotype of BDCA-1^+^ cDC or BDCA-3^+^ cDC, which was similar with T-VEC (Figure 4A). Analysis of the culture supernatant revealed a significantly greater FLT3L secretion upon treatment with oHSV1-FLT3L compared with untreated or T-VEC-treated BDCA-1^+^/BDCA-3^+^ cDCs, indicating that BDCA-1^+^/BDCA-3^+^ cDCs were infected by oHSV1-FLT3L (Figure 4B). There were no significant changes for the other cytokines, beside a trend toward a higher level of IFN-λ1, indicating that oHSV1-FLT3L or T-VEC itself did not or only marginally activated BDCA-1^+^/BDCA-3^+^ cDCs.

Given that we noticed that BDCA-1^+^/BDCA-3^+^ cDCs were infected by oHSV1-FLT3L, we assessed whether the virus is cytotoxic for BDCA-1^+^/BDCA-3^+^ cDCs by analyzing their viability using flow cytometry at different time points (24, 48, and 72 h) after infection. The viability of BDCA-3^+^ cDC1 remained stable across all conditions over time. In contrast, BDCA-1^+^ cDC showed a decrease in viability over time, which was, however, similar in the untreated and the oHSV1-FLT3L condition (Figure S7). Thus, we did not observe a cytotoxic effect on BDCA-1^+^/BDCA-3^+^ cDCs induced by oHSV1-FLT3L specifically.

### oHSV1-FLT3L-induced oncolysates induce partial activation of BDCA-1^+^/BDCA-3^+^ cDCs

Next, we sought to determine whether oHSV1-FLT3L-induced oncolysates are immunogenic and capable of inducing cDC maturation. For these experiments, we used both melanoma, both glioblastoma, and the two most OV-susceptible pancreatic ductal adenocarcinoma (BXPC3 and ASPC1) cell lines. To assess the cDC phenotype and functionality upon oHSV1-FLT3L treatment, we cultured purified BDCA-1^+^/BDCA-3^+^ cDCs along with the supernatant of cancer cell lines treated with oHSV1-FLT3L (MOI of 1) during 24 or 48 h. After 24 h of co-culture, the cDC phenotype was assessed by flow cytometry for expression of CD40, CD80, CD83, CD86, CD274, and HLA-ABC. The supernatant of these cultures was analyzed for their cytokine content (IL-10, IL-12p70, TNF-α, IFN-α-2a, IFN-λ1, and FLT3L).

**Figure 5:**
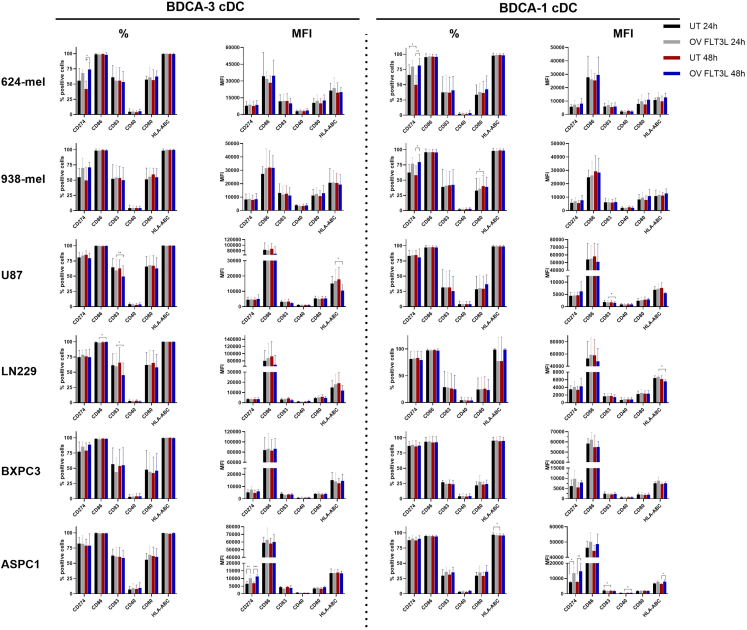
Effect of oHSV1-FLT3L-induced oncolysates on cDC phenotype Purified BDCA-1^+^/BDCA-3^+^ cDCs were co-cultured with supernatant of 624-mel, 938-mel, U87, LN229, BXPC3, and ASPC1 cell lines that were treated for 24 and 48 h with or without oHSV1-FLT3L (MOI of 1). The phenotype of BDCA-1^+^/BDCA-3^+^ cDCs was assessed upon analysis of DC maturation markers (CD274, CD86, CD83, CD40, CD80, and HLA-ABC) via flow cytometry after 24 h co-culture with oncolysates. Expression (% positive cells) as well as expression level (MFI) of every marker was displayed for BDCA-3^+^ cDC (left) and BDCA-1^+^ cDC (right). Bars represent mean ± SD of four independent experiments. Statistical analysis was performed with a repeated measures two-way ANOVA. Significance is indicated as ∗*p* ≤ 0.05, ∗∗*p* ≤ 0.01. MFI, mean fluorescence intensity; OV FLT3L, oncolytic virus oHSV1-FLT3L; UT, untreated.

On the phenotypic level, we observed minor changes upon co-culture of BDCA-1^+^/BDCA-3^+^ cDC with oHSV1-FLT3L-derived oncolysates (Figure 5). Co-culture with 624-mel oncolysates induced an upregulation of CD274-expressing cells, both for BDCA-1^+^ and BDCA-3^+^ cDCs, while co-culture with 938-mel oncolysates only induced upregulation of CD274-expressing cells in the BDCA-1^+^ cDC population. In co-cultures with ASPC1 oncolysates, both BDCA-1^+^ and BDCA-3^+^ cDCs showed an increased expression level of CD274, while BDCA-1^+^ cDC increased the expression level of HLA-ABC. In contrast, oncolysates of both glioblastoma and BXPC3-treated cell lines did not induce significant differences in the expression levels of maturation markers in BDCA-1^+^/BDCA-3^+^ cDCs as an effect of oHSV1-FLT3L-induced oncolysates.

**Figure 6:**
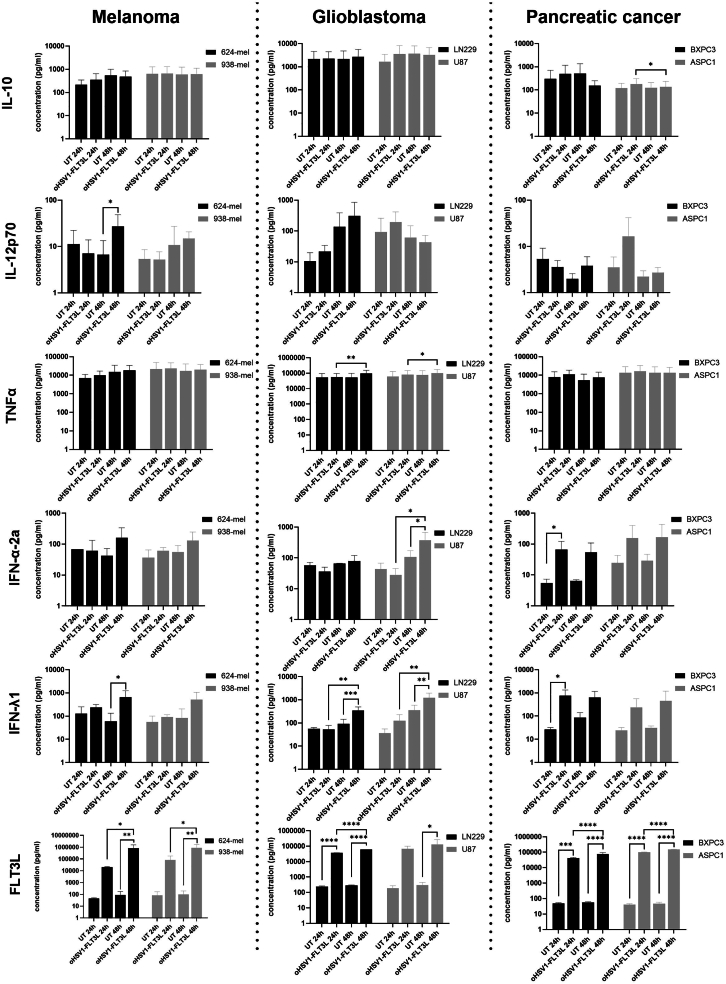
Effect of oHSV1-FLT3L-induced oncolysates on cDC cytokine secretion Purified BDCA-1^+^/BDCA-3^+^ cDCs were co-cultured with supernatant of 624-mel, 938-mel, U87, LN229, BXPC3, and ASPC1 cell lines that were treated for 24 and 48 h with or without oHSV1-FLT3L (MOI of 1). Cytokine secretion in the supernatants from these co-cultures was analyzed using a custom U-plex MesoScale Diagnostics assay for IL-10, IL-12p70, TNF-α, IFN-α-2a, IFN-λ1, and FLT3L. The graphs represent mean values ± SD from four independent experiments, except for ASPC1, which represents mean values ± SD from three independent experiments. Each sample was tested in duplicate. Undetectable values were substituted by the low detection limit/sqrt (2). For values above the detection limit of the assay, the highest detection limit was used. All data were statistically analyzed with a repeated measures two-way ANOVA. Statistical significance is indicated as ∗*p* ≤ 0.05, ∗∗*p* ≤ 0.01, ∗∗∗*p* ≤ 0.001, ∗∗∗∗*p* ≤ 0.0001. UT, Untreated.

We then analyzed the supernatant from these oncolysate-cDC co-cultures for cytokine secretion to assess the functional maturation of BDCA-1^+^/BDCA-3^+^ cDCs upon oHSV1-FLT3L treatment. The cytokine secretion profile showed more oHSV1-FLT3L-induced differences, although there was a high variability among donors (Figure 6). IL-10 levels were high and remained stable across all conditions. IL-12p70 levels were low and increased upon co-culture with oncolysates from 624-mel (48 h), 938-mel (48 h), LN229 (48 h), U87 (24 h), and ASPC1 (24 h), but this only reached significance for 624-mel. TNF-α concentrations were very high, but did not show variability across conditions. IFN-α-2a was detected at low levels and showed a trend toward increase upon addition of oHSV1-FLT3L for 624-mel (48 h), U87 (48 h), BXPC3 (24 h and 48 h), and ASPC1 (24 h and 48 h); however, significance could only be shown for U87 (48 h) and BXPC3 (24 h). Moderate levels of IFN-λ1 were measured in cDC supernatants, which increased upon addition of oHSV1-FLT3L-induced oncolysates from all tumor cell lines (48 h) and also using BXPC3 and ASPC1 oncolysates treated with oHSV1-FLT3L for 24 h. However, significance was only detected for 624-mel, LN229, U87, and BXPC3. Moderate FLT3L levels were measured in co-cultures with oncolysates from untreated tumor cell lines, which significantly increased in co-cultures with oHSV1-FLT3L oncolysates in all conditions, reflecting the presence of FLT3L from the oncolysates.

Altogether, these data showed partial BDCA-1^+^/BDCA-3^+^ cDC maturation upon co-culture with oHSV1-FLT3L-derived oncolysates, with minor phenotypic changes and more profound differences in their cytokine secretion pattern.

### Treatment with oHSV1-FLT3L induces ATP release by 938-mel, BXPC3, and ASPC1 cells

**Figure 7:**
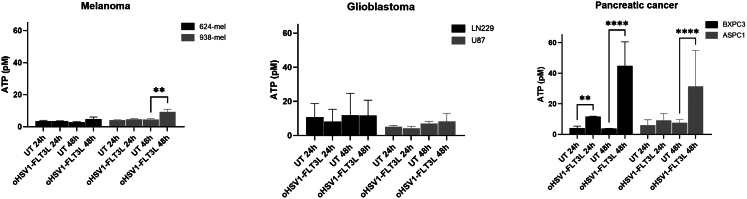
ATP release is induced by oHSV1-FLT3L in 938-mel and pancreatic cancer cells Tumor cells were treated with oHSV1-FLT3L at MOI 1 for 24 and 48 h. ATP concentrations were measured in the supernatants using a luminescence-based ATP determination kit. Data represent mean ± standard deviation of two independent experiments. All data were statistically analyzed with a repeated measures two-way ANOVA. Statistical significance is indicated as ∗∗*p* ≤ 0.01, ∗∗∗∗*p* < 0.0001. UT, Untreated.

In an attempt to investigate whether the partial maturation of cDCs upon co-culture with oHSV1-FLT3L-induced oncolysates was due to the release of damage-associated molecular patterns (DAMPs), we measured the release of HMGB1 and ATP upon treatment of tumor cell lines with oHSV1-FLT3L for 24 h and 48 h. HMGB1 levels for all cell lines and conditions were below the detection limit (1 ng/mL) of the assay (data not shown). The levels of released ATP were variable between cell lines. Pancreatic cancer cell lines showed the highest levels of ATP release. Upon 48 h of oHSV1-FLT3L treatment, we observed a significant increase of ATP release for 938-mel, BXPC3, and ASPC1 (Figure 7). However, we could not find a correlation between the levels of ATP in the supernatant and changes in phenotype or cytokine secretion by BDCA-1^+^/BDCA-3^+^ cDC induced by the oncolysates.

## Discussion

In this study, we report the design of an FLT3L-encoding oncolytic HSV1 that is capable of inhibiting the growth of melanoma, glioblastoma, and pancreatic adenocarcinoma cell lines and accompanied by the secretion of FLT3L. Differences in response to oHSV1-FLT3L infection by different cancer cell lines could be explained by differential expression of HSV entry receptor genes. However, it is important to underline that, across 11 different cell lines representative of 3 different cancer types, only 1 cell line (MiaPaca2) appears to be less sensitive to oHSV1-FLT3L-mediated growth inhibition. This suggests that resistance to this oHSV1 backbone in human cancer cells might be a relatively rare phenomenon. In addition, oHSV1-FLT3L-induced oncolysates are immunogenic and mediate partial activation of BDCA-1^+^/BDCA-3^+^ cDCs. These promising findings suggest the need for further investigation using more physiological 3D patient-derived tumor models^26^ and mouse models. These models would allow for a deeper exploration of the interactions between tumor cells and the components of the TME, such as immune cell infiltration, the presence of a fully functional immune system, and the impact of physical barriers within the TME. Additionally, studies should incorporate combinatorial treatments, including maturation-inducing agents, PD-L1 blockers, and regulatory T cell (Treg)-inhibiting agents.

Our data provide evidence for the development of oHSV1-FLT3L for the treatment of melanoma, glioblastoma, and pancreatic carcinoma. We have previously reported similar results for T-VEC in melanoma cell lines,^17^ although we observed that T-VEC was slightly more effective at inhibiting cancer cell growth in some of the tested cell lines, which might be due to the higher virulence of the clinical JS-1 strain in T-VEC compared with the 17syn^+^ strain used here. We observed a high variability in susceptibility to oHSV1-FLT3L infection between the distinct cancer cell lines and found that there was a correlation between the inhibition of tumor cell growth and the expression of *NECTIN2*, *ITGB6*, and *NECTIN1* genes. Previous studies have shown a strong association between sensitivity to T-VEC and nectin-1 expression in melanoma^27^^,^^28^ and a weak correlation in pancreatic cancer.^26^ Nectin-2 has been described as weak receptor for HSV1, but highly specific for HSV2, and it has been described that HSV1 OVs can infect tumor cells expressing only nectin-2, but that cell-to-cell spread is impaired compared with tumor cells expressing nectin-2 in combination with other entry receptors.^29^ The *ITGB6* gene encodes for the integrin subunit beta 6, which forms a dimer with integrin alpha V to form the integrin αvβ6. The αvβ6 integrin has been described as a receptor for the HSV gH/gL glycoprotein, promoting viral entry by the endocytic pathway. It has been documented that αvβ6 integrin can be upregulated in epithelial malignancies, favoring HSV infection.^30^^,^^31^^,^^32^ The correlation between inhibition of tumor cell growth and the expression of *NECTIN2*, *ITGB6*, and *NECTIN1* genes warrants their further investigation as potential biomarkers for oHSV1-FLT3L therapy.

One of the reasons by which OVs are thought to replicate specifically in tumor cells is due to deficiencies in antiviral machinery pathways. Indeed, it has been documented that cGAS, IFI16, and STING play a role in the detection of DNA viruses, while PKR and RIG-I are able to detect the dsRNA intermediate formed during transcription of the viral genome, which also results in STING activation.^33^ The γ34.5 neurovirulence gene product binds STING and prevents interaction with downstream effectors. Because the γ34.5 gene is deleted in T-VEC and oHSV1-FLTL3L, it is hypothesized that tumors with defects in the STING signaling axis are amenable to treatment with γ34.5-deleted oHSV. In our study, we could not find a correlation between *STING1* expression and sensitivity to infection by oHSV1-FLT3L, which is contradictory to other reports for T-VEC.^28^ However, when looking at the melanoma and glioblastoma cell lines, the highly sensitive U87 cell line did not express *STING1* or *CGAS*, which could explain its sensitivity. Both melanoma cell lines and the LN229 glioblastoma cell line expressed *STING1*, but did not express *CGAS*, which could again explain their sensitivity. This might suggest that looking at a single gene in the pathway is not enough and the ratio between different mediators in the signaling pathway might be important to determine the sensitivity of cells to infection with oHSV1-FLT3L. This was also suggested by Lipatova et al.,^34^ who stated that the suppression of a single gene encoding the most important components of the antiviral response to type I IFNs does not necessarily lead to an increase in the sensitivity of cells to OVs. In addition, we investigated gene expression levels, which does not necessarily result in protein expression, so it would be worth investigating the antiviral machinery at protein level. The picture was even more complex for the pancreatic cancer cell lines, since we clearly distinguished two phenotypes: one group expressing high levels of most investigated antiviral genes (BXPC3, ASPC1, SW1990, and Capan-1) and another group expressing only a few antiviral genes (PaTu8988t, SUIT2, and MiaPaca-2). Surprisingly, the group with the highest expression levels of antiviral genes was most sensitive to infection with oHSV1-FLT3L. Monsurrò et al.^35^ previously described that the antiviral state segregates two molecular phenotypes of pancreatic adenocarcinoma where the cell lines with high expression of antiviral genes were resistant to adenovirus infection. Furthermore, other studies have also reported that not all pancreatic cancer cell lines exhibit functional defects in the IFN signaling pathway. Buijs et al ^36^ and Moerdyk-Schauwecker et al.^37^ found that, unlike VSV, the infectivity and cytotoxicity of vaccinia and HSV in pancreatic cancer cells were independent of the type I IFN signaling profile. This suggests that these viruses may be better equipped to evade type I IFN responses.^36^^,^^37^

For OVs to stimulate effective anti-tumor immune responses, the mode of cell death induced by OVs should be immunogenic. To address this, we evaluated the effect of oHSV1-FLT3L itself and the effect of oHSV1-FLT3L-induced oncolysates on purified BDCA-1^+^/BDCA-3^+^ cDCs. The effects we observed were comparable to our previous data with T-VEC and those reported by Kalus et al.,^17^^,^^38^ although the effects with T-VEC seemed to be somewhat more pronounced.^17^^,^^38^ The fact that we were only able to detect one marker of ICD (ATP) but not HMGB1 invites further investigation into the mode of cell death induced by the viral vector. It is noteworthy that OVs can also be engineered to trigger ICD, for example, via the expression of pore proteins that mediate the efflux of cytoplasmic content from infected cells.^39^

Additionally, we observed that BDCA-1^+^/BDCA-3^+^ cDCs cultured with oHSV1-FLT3L led to the secretion of high levels of FLT3L compared with the untreated condition, indicating that BDCA-1^+^/BDCA-3^+^ cDCs were infected by the OV. This observation urged us to investigate whether this was accompanied by cytotoxic effects on the BDCA-1^+^/BDCA-3^+^ cDCs, but we did not observe differences in viability between untreated and oHSV1-FLT3L-treated cDCs up to 72 h of culture, indicating that OV infection of BDCA-1^+^/BDCA-3^+^ cDCs did not induce cell death.

CD274 or PD-L1 showed the most pronounced upregulation on BDCA-1^+^/BDCA-3^+^ cDCs, both with oHSV1-FLT3L and T-VEC. This might point to the phenotype of mature DCs enriched in immunoregulatory molecules (mregDCs), an immunoregulatory program that is expressed by cDC1 and cDC2 upon the uptake of tumor antigens and restrains DC immunostimulatory function and controls the threshold of T cell activation.^40^ In addition, Clappaert et al.^41^ showed that these mregDCs (or migratory DCs) are increased in tumors upon systemic FLT3L treatment, and especially a CD81-expressing mregDC1 population with potent Treg-inducing properties. However, their data also show that FLT3L treatment is unable to induce anti-tumor immunity and reduce tumor growth in the murine cancer setting. To our knowledge, these data have not yet been reported in cancer patients, and FLT3L-containing treatment regimens have shown to induce anti-tumor immune responses in solid and hematological tumors.^42^^,^^43^ However, the data from Clappaert et al.^41^ warrant a thorough investigation of the DC subsets and the subsequent anti-tumor immune responses induced by FLT3L in cancer patients in order to clarify the utility of FLT3L treatment as a component of cancer therapies.

We observed that both IFN-α-2a and IFN-λ1 are upregulated upon co-culture of cDCs with oHSV1-FLT3L-induced oncolysates. Both IFN-α-2a and IFN-λ1 play a role in viral infections, so it could be possible that they are upregulated as a consequence of sensing of viral PAMPs. However, type I IFN (IFN-α-2a) and type III IFN (IFN-λ1) have been shown to have important anti-tumor effects beside their antiviral activity.^44^^,^^45^ In human tumors, IFN-λ1 is selectively produced by cDC1, which is enhanced by poly(I:C) stimulation, and it is associated with a favorable clinical outcome,^46^^,^^47^ possibly by inducing a T helper type 1-oriented TME.

In conclusion, we developed an oHSV1-FLT3L that is capable of inhibiting the growth of melanoma, glioblastoma, and pancreatic cancer cells, simultaneously inducing FLT3L secretion. The growth inhibitory effect was correlated to the expression of *NECTIN1*, *NECTIN2*, and *ITGB6* by the tumor cell lines. Oncolysates induced by oHSV1-FLT3L induced partial BDCA-1^+^/BDCA-3^+^ cDC maturation, probably mediated by the presence of viral PAMPs and immunogenic DAMPs resulting from ICD.

## Materials and methods

### Cell lines

The human-derived melanoma cell lines 624-mel and 938-mel were a kind gift from Prof. S. Topalian (Institute for Cancer Immunotherapy, The Johns Hopkins University School of Medicine). Human glioblastoma cell lines LN229 and U87 and pancreatic cancer cell lines BXPC3, ASPC1, SW1990, Capan-1, PaTu8988t, SUIT2, and MiaPaca-2 were purchased from ATCC. Vero CCL81 cells were a gift from Prof. Aerts (VUB, NAVI). Specific cell culture media are described in Table S1. Cells were cultured at 37°C and 5% CO_2_, and cultures were regularly tested for the absence of *Mycoplasma* contamination.

### Generation of oHSV1-FLT3L

A synthetic sequence encoding the cDNA of the human *FLT3LG* gene was obtained from Biofab Research cloned within a pUC57 plasmid. Starting from a previously developed strain 17+ oHSV1 with deletions of both copies of the *γ34.5* neurovirulence gene and of the *US12* gene,^25^ the EGFP-expressing cassette in the viral *UL55-UL56* intergenic region was replaced by bacterial artificial chromosome (BAC) mutagenesis with a similar eukaryotic expression cassette in which the expression of FLT3L was driven by the immediate-early cytomegalovirus promoter/enhancer. The original 17+ HSV-1 BAC including γ34.5 deletions was kindly provided by Prof. Beate Sodeik (Hannover Medical School). Importantly, the deletions introduced in the viral genome mirror those found in the clinically available T-VEC. Thus, an oHSV1-FLT3L BAC was produced. BAC mutagenesis, viral reconstitution, and stock production were performed as previously described.^48^ A visual representation of the viral vector is shown in Figure S8.

### Viral amplification and titration

Viral amplification was performed as described previously.^48^ Briefly, Vero cells were seeded and incubated at 37°C. The following day, the cells were infected with the viral mix, a viral stock of oHSV1-FLT3L at an MOI of 0.01 plaque-forming units (PFU)/cell diluted in serum-free DMEM (Thermo Fisher Scientific) and incubated for 1 h at 37°C. Upon removal of the viral mix, the cells were incubated with DMEM 2% FBS (Greiner Bio-One), 10,000 U/mL penicillin/streptomycin (Thermo Fisher Scientific) and 2 μM L-glutamine (Thermo Fisher Scientific) and incubated at 37°C for at least 72 h. From 72 h post infection, the amplification can be blocked. Four cycles of freezing, thawing, and sonication were performed to break the cell membrane and release the virus. The supernatant was aliquoted and stored at −80°C.

Upon viral amplification, a plaque assay was performed to determine the titer of the amplified virus stock.^48^ Briefly, Vero cells were plated and incubated overnight at 37°C. The next day, serial viral dilutions (10^−3^, 10^−4^, 10^−5^, 10^−6^, and 10^−7^) were added and incubated for 1 h at 37°C. After virus removal, DMEM (Thermo Fisher Scientific) supplemented with 2% FBS (Greiner Bio-One) and 0.75% methyl cellulose (Merck Life Science) was added and incubated at 37°C. Seventy-two hours post infection, viral plaques were formed, and cells were fixed with 4% formaldehyde (Merck Life Science) at room temperature (RT) for 10 min. Upon removal of formaldehyde, crystal violet (Merck Life Science) was added and incubated at RT for 5 min. After removal, the plate was left to dry, and plaques were counted (between 5 and 50 isolated plaques). Calculation of the viral titer was done with the following formula: PFU/mL = (plaques average × dilution factor)/infection volume.

### Incucyte proliferation and cytotoxicity assay

Human cancer cell lines were seeded in flat-bottom 96-well plates (Greiner Bio-One) at a density of 5 × 10^3^ cells per well (PaTu8988t and SUIT2), 1 × 10^4^ cells per well (624-mel, 938-mel, LN229, U87, BXPC3, ASPC1, MiaPaca-2, and SW1990), and 2 × 10^4^ cells per well (Capan-1) and left to adhere overnight at 37°C, 5% CO_2_. The following day, oHSV1-FLT3L was added at different MOIs (0.001, 0.01, 0.1, 1, or 10 PFU/cell) in triplicate, and cells were incubated in the Incucyte SX5 instrument (Sartorius). Cell growth was monitored continuously with a 10× objective every 2 h over a period of a maximum of 170 h. Cell confluency (%) over time was analyzed using the Incucyte analysis software version 2023A (Sartorius). Upon normalization of every time point to baseline, the AUC was calculated as a mean of the triplicates per MOI, which was then normalized to blank (untreated cells). To categorize the cell lines by their susceptibility to oHSV1-FLT3L-mediated growth inhibition, we first calculated the sum of all AUCs at the various MOIs tested. This sum was then multiplied by the rank of the lowest MOI at which confluence significantly differed from the untreated condition (with ranks ranging from 1 for MOI 0.001 to 5 for MOI 10, and 6 for cases where no significant differences in AUC were observed). The resulting value was lowest for the most susceptible cell line and increased as susceptibility decreased.

To assess the induction of cell death, the Incucyte cytotox red dye (Sartorius) was added at a concentration of 250 nM to the wells at the time of OV addition, and red fluorescent signal was measured continuously with a 10× objective every 2 h over a period of a 96 h. The number of dead cells per square millimeter was quantified using the Incucyte analysis software version 2023A. The number of dead cells per square millimeter was then normalized to the cell confluency, after which the AUC was calculated.

### FLT3L ELISA

Cancer cell lines were plated on day 0 in identical conditions as the Incucyte proliferation assay and left to adhere overnight at 37°C, 5% CO_2_. At day 1, oHSV1-FLT3L was added as described for the Incucyte proliferation assay and cells were incubated. Supernatant was collected after 8, 24, 48, 72, and 120 h of incubation time upon oHSV1-FLT3L treatment and stored at −20°C for further use. Release of FLT3L upon treatment with oHSV1-FLT3L was measured in the collected supernatant and compared with control (untreated cells) with a DuoSet ELISA human FLT3L kit (Bio-Techne), according to manufacturer’s instructions. Supernatants of OV-treated cell lines were tested in triplicate and at different dilutions. Optical density (OD) values were measured with an xMark Microplate spectrophotometer (Bio-Rad Laboratories, Temse, Belgium) using the MPM6 software at a wavelength of 450 nm (with a correction at 570 nm). FLT3L concentration in supernatants was calculated using a standard curve (4PL curve fit). Data are displayed as mean ± SD of triplicate wells.

### Gene expression analysis

From all cancer cell lines, RNA was isolated in triplicate using the NucleoSpin RNA Plus kit (Macherey-Nagel) following the manufacturer’s protocol. NanoDrop (Thermo Fisher Scientific) was performed to determine RNA concentration and purity (260/280 absorbance ratio). After quality control of the RNA samples using an Agilent Bioanalyzer RNA 6000 Nano assay (Agilent Technologies), RNA libraries were prepared and subjected to rRNA-depleted RNA sequencing (Watchmaker Genomics). After quality control and read alignment to the reference genome, raw read counts were normalized using DESeq2 and log transformed (R version 4.4.1). Normalized gene expression levels were scaled and plotted in a heatmap (pheatmap version 1.0.12). Correlations between gene expression levels and the sensitivity of the cell lines to oHSV1-FLT3L-mediated growth inhibition (calculated as described under Incucyte proliferation assay) were assessed by calculating the Pearson and Spearman correlation coefficients.

### Isolation and culture of BDCA-1^+^/BDCA-3^+^ conventional cDCs

Isolated BDCA-1^+^/BDCA-3^+^ cDCs were obtained from patients included in various clinical trials (NCT04571632, NCT03233152, and NCT03707808) at UZ Brussel, from whom excess cDCs were available for translational research. These studies have been conducted in accordance with the Declaration of Helsinki and were approved by the ethics committee of the UZ Brussel. Patients provided written informed consent to use cells that were not used for treatment for research purposes. BDCA-1^+^/BDCA-3^+^ cDCs were isolated and characterized as previously described.^17^

Purified BDCA-1^+^/BDCA-3^+^ cDCs were resuspended in X-VIVO-15 medium (Lonza) supplemented with 10,000 U/mL penicillin/streptomycin (Thermo Scientific), 2 μM L-glutamine (Thermo Scientific), 100 mM sodium pyruvate (Thermo Scientific), and GM-CSF (Miltenyi Biotec; final concentration of 10^5^ IU/mL) (DC medium). Cells were plated at 2 × 10^5^ cells per well in 100 μL in an ultra-low attachment 96-well plate. Addition of an equal volume of DC medium is used as a negative control, and DC medium with R848 (1 mg/mL, Invivogen) with poly(I:C) (20 μg/mL, Invivogen) as a positive control. DC medium with oHSV1-FLT3L at an MOI of 1 or supernatant of oHSV1-FLT3L-treated cancer cell lines (100 μL) was added and incubated overnight (37°C, 5% CO_2_). After incubation, 150 μL of this co-culture supernatant was collected and stored at −20°C for further cytokine analysis. The cells were resuspended in PBS and used for flow cytometry. For viability assessment, the same collection was done upon 24, 48, and 72 h of incubation.

### Flow cytometry

Cells were centrifuged and incubated with TruStain FcX (BioLegend) to block Fc receptors, after which the samples were stained with a prepared antibody mix. For maturation assessment, the following monoclonal antibodies were used: CD11c Alexa Fluor 700 (BD Biosciences, clone B-ly6), CD1c BV510 (BD Biosciences, clone F10/21A3), CD141 PE-Cy7 (BioLegend, clone JAA17), CD274 PE-Dazzle594 (BioLegend, clone MIH1), CD86 BV421 (BD Biosciences, clone 2331 (FUN-1)), CD83 PE (BD Biosciences, clone HB15e), CD40 APC (BD Biosciences, clone 5C3), CD80 PE-Cy5 (BD Biosciences, clone L307.4), HLA-ABC FITC (BD Biosciences, clone G46–2.6), and zombie yellow (BioLegend) for dead cell exclusion. Antibodies used for viability staining were the following: CD11c Alexa Fluor 700 (BD Biosciences, clone B-ly6), CD1c BV510 (BD Biosciences, clone F10/21A3), CD141 PE-Cy7 (BioLegend, clone JAA17), CD14 FITC (BioLegend, clone HCD14), CD45 PE (Miltenyi Biotec, clone REA747), FcεR Vioblue (Miltenyi Biotec, clone CRA1) for DC gating, and 7-Aminoactinomycin D (7-AAD, Thermo Fisher Scientific) for dead cell assessment. Samples were incubated for 20 min at 4°C and resuspended in PBS (Thermo Fisher Scientific) before acquisition on LSR Fortessa or FACSSymphony cytometer (BD Biosciences) where single-stained controls were used for compensation. Data analysis was performed with FlowJo version 10.10.0. The gating strategies are shown in Figure S9.

### Multiplex cytokine analysis

Supernatant from BDCA-1^+^/BDCA-3^+^ cDC co-cultures with oHSV1-FLT3L-induced oncolysates was analyzed for cytokine content. The levels of IL-10, IL-12p70, TNF-α, IFN-α-2a, IFN-λ1, and FLT3L were analyzed using a human custom U-plex assay (Meso Scale Diagnostics) according to the manufacturer’s instructions. Light intensity was measured with a MESO QuickPlex SQ 120 instrument (Meso Scale Diagnostics) and analyzed via the MSD Discovery Workbench software version 4. For samples below fit curve range, the following substitute was used: (assay detection limit)/sqrt (2).

### Quantification of ATP and HMGB1

Cancer cell lines (624-mel, 938-mel, LN229, U87, BXPC3, and ASPC1) were plated at a density of 1 × 10^4^ cells per 96-well and infected with oHSV1-FLT3L at MOI 1 for 24 or 48 h, after which supernatant was harvested and stored at −20°C. ATP levels were determined using an ATP determination kit (Thermo Fisher Scientific), following the manufacturer’s instructions. Luminescence was measured using the GloMax microplate reader (Promega). Data were analyzed by interpolating a hyperbolic standard curve in GraphPad Prism version 10.1.1. HMGB1 was measured using a HMGB1 ELISA (Tecan), following the manufacturer’s instructions. OD values were measured with an xMark Microplate spectrophotometer (Bio-Rad) via the MPM6 software at a wavelength of 450 nm (with correction at 650 nm).

### Statistical analysis

All statistical analyses were performed in GraphPad Prism version 10.1.1. software. For Incucyte data, the untreated condition was compared with the oHSV1-FLT3L-treated group at different MOIs using an ordinary one-way ANOVA with Dunnett’s multiple comparisons test. FLT3L secretion was analyzed using a mixed-effects model and cell viability using a repeated measures two-way ANOVA with Tukey’s multiple comparisons test, each time assessing the effects of time and MOI and their interaction. Repeated measures two-way ANOVA with uncorrected Fishers least significant difference and single pool variance was performed to assess the effects of the treatment (untreated vs. oHSV1-FLT3L) and time (24 h vs. 48 h) for each condition of cDC maturation as well as cytokine analysis. Differences were considered statistically significant from a *p* value of ≤0.05.

## Data availability

The RNA sequencing data are freely available at GSE288221. All other data are available upon reasonable request. All data relevant to the study are included in the article or uploaded as supplemental information.

## Acknowledgments

The authors would like to thank Simona Nedyalkova, Fiona Doumbouya, and Anisa Aliju for their assistance in the experimental work. Furthermore, Hugo Vandenplas and Maya Verdeye are acknowledged for technical assistance.

This work was supported by the “Wetenschappelijk Fonds Willy Gepts” from the Vrije Universiteit Brussel. X.G. received a personal postdoctoral mandate from the Foundation Against Cancer (Stichting tegen Kanker). The UMCOR Incucyte Core Facility (VUB) that was used for this work received financial support of the University Medical Center Research Council (UMCOR), while the FlowCore Research facility was supported by the VUB Research Council (Dutch: onderzoeksraad, OZR, grant ID: OZR3715). Graphical abstract was created in BioRender: Tuyaerts, S. (2025) https://BioRender.com/2j4re9o. The funders had no role in the study design, data collection, analysis, interpretation, writing of the manuscript, or decision to submit it for publication.

## Author contributions

S.T., X.G., A.R., G.B., and I.V.R. conducted the experiments. S.T., X.G., and L.S contributed to the study design, data analysis, and data interpretation. J.B. and T.J. contributed to the bioinformatics analysis. S.T. drafted the manuscript and S.T., X.G., A.R., L.S., A.C., and B.N. contributed the critical revision of the manuscript. All authors approved the final submitted version of the manuscript.

## Declaration of interests

B.N. has received honoraria for public speaking or advisory board participation from Roche, Bristol-Myers Squibb, MSD, Novartis, AstraZeneca, and Miltenyi Biotec.

## Supplemental information

Document S1. Figures S1–S9 and Tables S1–S5

Document S2. Article plus supplemental information
