# Supplementary material for: Oncolytic HSV-1 expressing FLT3L kills melanoma, glioblastoma, and pancreatic cancer cells in vitro and induces immunogenic cell death
Source: Mol Ther Oncol. 2025 Aug 9;33(3):201031. doi: 10.1016/j.omton.2025.201031 (PMC12433480; doi:10.1016/j.omton.2025.201031)
Supplement: Document S1. Figures S1–S9 and Tables S1–S5 [file mmc1.pdf]

OMTON, Volume *33*

Supplemental information

Oncolytic HSV-1 expressing FLT3L kills melanoma, glioblastoma, and pancreatic cancer cells *in vitro* and induces immunogenic cell death

Sandra Tuyaerts, Xenia Geeraerts, Alberto Reale, Latoya Stevens, Giada Bertazzon, Jack Brons, Toon Janssen, Ivan Van Riet, Arianna Calistri, and Bart Neyns

Supplemental tables

Table S1: Time-dependent p-values statistical analysis FLT3L ELISA

	Time-dependent	untreated	0,001	0,01	0,1	1	10
**624-mel**	24 vs. 48	>0,9999	0,9947	<0,0001	<0,0001	<0,0001	0,9459
	24 vs. 72	>0,9999	<0,0001	<0,0001	<0,0001	<0,0001	0,9915
	48 vs. 72	>0,9999	<0,0001	<0,0001	0,7099	0,9788	0,9124
**938-mel**	24 vs. 48	0,9916	0,8555	<0,0001	<0,0001	0,1076	0,9845
	24 vs. 72	0,9988	<0,0001	<0,0001	<0,0001	0,1493	0,9956
	48 vs. 72	0,9978	<0,0001	<0,0001	0,8798	0,9987	0,998
**LN229**	24 vs. 48	>0,9999	0,9998	0,0104	<0,0001	<0,0001	0,8583
	24 vs. 72	>0,9999	0,9979	<0,0001	<0,0001	<0,0001	0,9868
	48 vs. 72	>0,9999	0,999	<0,0001	0,9027	0,6939	0,7752
**U87**	24 vs. 48	0,9992	0,9975	0,5854	<0,0001	<0,0001	0,3538
	24 vs. 72	>0,9999	0,9994	<0,0001	<0,0001	<0,0001	0,9946
	48 vs. 72	0,999	0,9989	<0,0001	>0,9999	0,3707	0,4042
**BXPC3**	24 vs. 48	0,9997	<0,0001	<0,0001	<0,0001	<0,0001	<0,0001
	24 vs. 72	0,9998	<0,0001	<0,0001	<0,0001	<0,0001	<0,0001
	48 vs. 72	>0,9999	0,0002	0,9101	0,9256	>0,9999	0,9152
**ASPC1**	24 vs. 48	>0,9999	0,9383	0,7265	0,0831	<0,0001	<0,0001
	24 vs. 72	>0,9999	0,0028	<0,0001	<0,0001	<0,0001	<0,0001
	48 vs. 72	>0,9999	0,0061	<0,0001	<0,0001	0,6032	0,9984
**SUIT2**	24 vs. 48	0,9892	0,9951	0,9776	0,1499	<0,0001	<0,0001
	24 vs. 72	0,9978	0,8078	0,0009	<0,0001	<0,0001	<0,0001
	48 vs. 72	0,9953	0,7356	0,0005	<0,0001	0,9752	0,7994
**Capan-1**	24 vs. 48	>0,9999	0,7769	0,7352	0,4161	<0,0001	<0,0001
	24 vs. 72	>0,9999	0,1852	0,1747	<0,0001	<0,0001	<0,0001
	48 vs. 72	>0,9999	0,5011	0,5236	<0,0001	<0,0001	<0,0001
**PaTu8988t**	24 vs. 48	>0,9999	0,9977	0,9544	0,0853	<0,0001	<0,0001
	24 vs. 72	>0,9999	0,4083	<0,0001	<0,0001	<0,0001	<0,0001
	48 vs. 72	>0,9999	0,4445	<0,0001	<0,0001	0,0651	0,9853
**SW1990**	24 vs. 48	>0,9999	>0,9999	0,3916	<0,0001	0,0051	0,4282
	24 vs. 72	>0,9999	0,5236	<0,0001	<0,0001	<0,0001	0,001
	48 vs. 72	>0,9999	0,5236	<0,0001	<0,0001	0,0004	0,0274
**MiaPaca2**	24 vs. 48	0,9794	>0,9999	>0,9999	0,9987	0,6115	0,0001
	24 vs. 72	0,9996	0,9997	0,9846	>0,9999	0,0034	<0,0001
	48 vs. 72	0,987	0,9994	0,9846	0,998	0,014	0,3699

Table S2: MOI-dependent p-values statistical analysis FLT3L ELISA

	MOI-dependent	24h	48h	72h
	untreated vs. MOI 0.001	>0,9999	>0,9999	<0,0001
	untreated vs. MOI 0.01	>0,9999	<0,0001	<0,0001
	untreated vs. MOI 0.1	0,9976	<0,0001	<0,0001
	untreated vs. MOI 1	<0,0001	<0,0001	<0,0001
	untreated vs. MOI 10.0	<0,0001	<0,0001	<0,0001
	MOI 0.001 vs. MOI 0.01	>0,9999	<0,0001	0,1411
	MOI 0.001 vs. MOI 0.1	0,9976	<0,0001	0,1088
**624-mel**	MOI 0.001 vs. MOI 1	<0,0001	<0,0001	0,1234
	MOI 0.001 vs. MOI 10.0	<0,0001	<0,0001	0,1309
	MOI 0.01 vs. MOI 0.1	0,9984	<0,0001	>0,9999
	MOI 0.01 vs. MOI 1	<0,0001	<0,0001	>0,9999
	MOI 0.01 vs. MOI 10.0	<0,0001	<0,0001	>0,9999
	MOI 0.1 vs. MOI 1	<0,0001	0,9909	>0,9999
	MOI 0.1 vs. MOI 10.0	<0,0001	0,8191	>0,9999
	MOI 1 vs. MOI 10.0	<0,0001	0,9871	>0,9999
	untreated vs. MOI 0.001	>0,9999	0,9982	<0,0001
	untreated vs. MOI 0.01	>0,9999	<0,0001	<0,0001
	untreated vs. MOI 0.1	0,5306	<0,0001	<0,0001
	untreated vs. MOI 1	<0,0001	<0,0001	<0,0001
	untreated vs. MOI 10.0	<0,0001	<0,0001	<0,0001
	MOI 0.001 vs. MOI 0.01	>0,9999	<0,0001	<0,0001
	MOI 0.001 vs. MOI 0.1	0,5354	<0,0001	<0,0001
**938-mel**	MOI 0.001 vs. MOI 1	<0,0001	<0,0001	<0,0001
	MOI 0.001 vs. MOI 10.0	<0,0001	<0,0001	<0,0001
	MOI 0.01 vs. MOI 0.1	0,6071	<0,0001	0,9996
	MOI 0.01 vs. MOI 1	<0,0001	<0,0001	0,9997
	MOI 0.01 vs. MOI 10.0	<0,0001	<0,0001	>0,9999
	MOI 0.1 vs. MOI 1	<0,0001	0,9967	>0,9999
	MOI 0.1 vs. MOI 10.0	<0,0001	0,9979	>0,9999
	MOI 1 vs. MOI 10.0	0,4297	>0,9999	>0,9999
	untreated vs. MOI 0.001	>0,9999	>0,9999	>0,9999
	untreated vs. MOI 0.01	>0,9999	0,0255	<0,0001
	untreated vs. MOI 0.1	0,8408	<0,0001	<0,0001
	untreated vs. MOI 1	<0,0001	<0,0001	<0,0001
	untreated vs. MOI 10.0	<0,0001	<0,0001	<0,0001
	MOI 0.001 vs. MOI 0.01	>0,9999	0,028	<0,0001
	MOI 0.001 vs. MOI 0.1	0,8631	<0,0001	<0,0001
**LN229**	MOI 0.001 vs. MOI 1	<0,0001	<0,0001	<0,0001
	MOI 0.001 vs. MOI 10.0	<0,0001	<0,0001	<0,0001
	MOI 0.01 vs. MOI 0.1	0,9006	<0,0001	>0,9999
	MOI 0.01 vs. MOI 1	<0,0001	<0,0001	0,9997
	MOI 0.01 vs. MOI 10.0	<0,0001	<0,0001	0,9794
	MOI 0.1 vs. MOI 1	<0,0001	0,9503	0,9993
	MOI 0.1 vs. MOI 10.0	<0,0001	0,9998	0,9625
	MOI 1 vs. MOI 10.0	<0,0001	0,9902	0,9969
	untreated vs. MOI 0.001	>0,9999	>0,9999	>0,9999
	untreated vs. MOI 0.01	>0,9999	0,9265	<0,0001
	untreated vs. MOI 0.1	0,8201	<0,0001	<0,0001
	untreated vs. MOI 1	<0,0001	<0,0001	<0,0001
	untreated vs. MOI 10.0	<0,0001	<0,0001	<0,0001
	MOI 0.001 vs. MOI 0.01	>0,9999	0,9836	<0,0001
	MOI 0.001 vs. MOI 0.1	0,8263	<0,0001	<0,0001
**U87**	MOI 0.001 vs. MOI 1	<0,0001	<0,0001	<0,0001
	MOI 0.001 vs. MOI 10.0	<0,0001	<0,0001	<0,0001
	MOI 0.01 vs. MOI 0.1	0,8425	<0,0001	0,8462
	MOI 0.01 vs. MOI 1	<0,0001	<0,0001	0,7063
	MOI 0.01 vs. MOI 10.0	<0,0001	<0,0001	0,3618
	MOI 0.1 vs. MOI 1	<0,0001	0,9142	0,9997
	MOI 0.1 vs. MOI 10.0	<0,0001	0,9996	0,9352
	MOI 1 vs. MOI 10.0	<0,0001	0,9806	0,987

	MOI-dependent	24h	48h	72h
	untreated vs. MOI 0.001	>0,9999	<0,0001	<0,0001
	untreated vs. MOI 0.01	0,9998	<0,0001	<0,0001
	untreated vs. MOI 0.1	0,7259	<0,0001	<0,0001
	untreated vs. MOI 1	<0,0001	<0,0001	<0,0001
	untreated vs. MOI 10.0	<0,0001	<0,0001	<0,0001
	MOI 0.001 vs. MOI 0.01	>0,9999	0,0036	>0,9999
	MOI 0.001 vs. MOI 0.1	0,7104	0,0008	0,9997
**BXPC3**	MOI 0.001 vs. MOI 1	<0,0001	0,0006	>0,9999
	MOI 0.001 vs. MOI 10.0	<0,0001	0,0006	>0,9999
	MOI 0.01 vs. MOI 0.1	0,7973	0,9955	0,9997
	MOI 0.01 vs. MOI 1	<0,0001	0,9906	>0,9999
	MOI 0.01 vs. MOI 10.0	<0,0001	0,9897	>0,9999
	MOI 0.1 vs. MOI 1	<0,0001	>0,9999	0,997
	MOI 0.1 vs. MOI 10.0	<0,0001	>0,9999	>0,9999
	MOI 1 vs. MOI 10.0	0,9963	>0,9999	0,9987
	untreated vs. MOI 0.001	>0,9999	0,9998	0,0073
	untreated vs. MOI 0.01	>0,9999	0,9926	<0,0001
	untreated vs. MOI 0.1	>0,9999	0,5746	<0,0001
	untreated vs. MOI 1	0,9977	<0,0001	<0,0001
	untreated vs. MOI 10.0	0,0212	<0,0001	<0,0001
	MOI 0.001 vs. MOI 0.01	>0,9999	0,998	0,0003
	MOI 0.001 vs. MOI 0.1	>0,9999	0,3865	<0,0001
**ASPC1**	MOI 0.001 vs. MOI 1	0,991	<0,0001	<0,0001
	MOI 0.001 vs. MOI 10.0	0,0005	<0,0001	<0,0001
	MOI 0.01 vs. MOI 0.1	>0,9999	0,6481	0,3901
	MOI 0.01 vs. MOI 1	0,9916	<0,0001	0,3604
	MOI 0.01 vs. MOI 10.0	0,0005	<0,0001	0,4499
	MOI 0.1 vs. MOI 1	0,9943	<0,0001	>0,9999
	MOI 0.1 vs. MOI 10.0	0,0006	<0,0001	>0,9999
	MOI 1 vs. MOI 10.0	0,0028	0,9745	>0,9999
	untreated vs. MOI 0.001	>0,9999	>0,9999	0,9754
	untreated vs. MOI 0.01	>0,9999	>0,9999	0,0019
	untreated vs. MOI 0.1	>0,9999	0,4468	<0,0001
	untreated vs. MOI 1	0,9378	<0,0001	<0,0001
	untreated vs. MOI 10.0	0,0068	<0,0001	<0,0001
	MOI 0.001 vs. MOI 0.01	>0,9999	>0,9999	0,0043
	MOI 0.001 vs. MOI 0.1	>0,9999	0,431	<0,0001
**SUIT2**	MOI 0.001 vs. MOI 1	0,9446	<0,0001	<0,0001
	MOI 0.001 vs. MOI 10.0	0,0073	<0,0001	<0,0001
	MOI 0.01 vs. MOI 0.1	>0,9999	0,4664	<0,0001
	MOI 0.01 vs. MOI 1	0,8511	<0,0001	<0,0001
	MOI 0.01 vs. MOI 10.0	0,0004	<0,0001	<0,0001
	MOI 0.1 vs. MOI 1	0,8309	<0,0001	0,9952
	MOI 0.1 vs. MOI 10.0	0,0001	<0,0001	0,9565
	MOI 1 vs. MOI 10.0	0,0031	>0,9999	0,9994
	untreated vs. MOI 0.001	>0,9999	0,9846	0,5291
	untreated vs. MOI 0.01	>0,9999	0,9762	0,5077
	untreated vs. MOI 0.1	>0,9999	0,8061	<0,0001
	untreated vs. MOI 1	0,6807	<0,0001	<0,0001
	untreated vs. MOI 10.0	<0,0001	<0,0001	<0,0001
	MOI 0.001 vs. MOI 0.01	>0,9999	>0,9999	>0,9999
	MOI 0.001 vs. MOI 0.1	>0,9999	0,991	0,0002
**Capan-1**	MOI 0.001 vs. MOI 1	0,7021	<0,0001	<0,0001
	MOI 0.001 vs. MOI 10.0	<0,0001	<0,0001	<0,0001
	MOI 0.01 vs. MOI 0.1	>0,9999	0,995	0,0002
	MOI 0.01 vs. MOI 1	0,7038	<0,0001	<0,0001
	MOI 0.01 vs. MOI 10.0	<0,0001	<0,0001	<0,0001
	MOI 0.1 vs. MOI 1	0,7194	<0,0001	<0,0001
	MOI 0.1 vs. MOI 10.0	<0,0001	<0,0001	<0,0001
	MOI 1 vs. MOI 10.0	<0,0001	<0,0001	0,9562

	MOI-dependent	24h	48h	72h
	untreated vs. MOI 0.001	>0,9999	>0,9999	0,864
	untreated vs. MOI 0.01	>0,9999	0,9997	<0,0001
	untreated vs. MOI 0.1	>0,9999	0,2529	<0,0001
	untreated vs. MOI 1	0,9988	<0,0001	<0,0001
	untreated vs. MOI 10.0	0,0612	<0,0001	<0,0001
	MOI 0.001 vs. MOI 0.01	>0,9999	0,9999	<0,0001
	MOI 0.001 vs. MOI 0.1	>0,9999	0,276	<0,0001
**PaTu8988t**	MOI 0.001 vs. MOI 1	0,9986	<0,0001	<0,0001
	MOI 0.001 vs. MOI 10.0	0,0594	<0,0001	<0,0001
	MOI 0.01 vs. MOI 0.1	>0,9999	0,3958	<0,0001
	MOI 0.01 vs. MOI 1	0,9987	<0,0001	<0,0001
	MOI 0.01 vs. MOI 10.0	0,0602	<0,0001	<0,0001
	MOI 0.1 vs. MOI 1	0,9991	<0,0001	0,9976
	MOI 0.1 vs. MOI 10.0	0,0648	<0,0001	>0,9999
	MOI 1 vs. MOI 10.0	0,1404	0,4353	0,9889
	untreated vs. MOI 0.001	>0,9999	>0,9999	0,8804
	untreated vs. MOI 0.01	>0,9999	0,7702	<0,0001
	untreated vs. MOI 0.1	0,9995	<0,0001	<0,0001
	untreated vs. MOI 1	0,0087	<0,0001	<0,0001
	untreated vs. MOI 10.0	<0,0001	<0,0001	<0,0001
	MOI 0.001 vs. MOI 0.01	>0,9999	0,7702	<0,0001
	MOI 0.001 vs. MOI 0.1	0,9995	<0,0001	<0,0001
**SW1990**	MOI 0.001 vs. MOI 1	0,0087	<0,0001	<0,0001
	MOI 0.001 vs. MOI 10.0	<0,0001	<0,0001	<0,0001
	MOI 0.01 vs. MOI 0.1	0,9995	0,0012	<0,0001
	MOI 0.01 vs. MOI 1	0,0087	<0,0001	0,1686
	MOI 0.01 vs. MOI 10.0	<0,0001	<0,0001	0,0072
	MOI 0.1 vs. MOI 1	0,0201	0,7664	0,0017
	MOI 0.1 vs. MOI 10.0	<0,0001	0,0019	0,0559
	MOI 1 vs. MOI 10.0	0,0002	0,0615	0,7687
	untreated vs. MOI 0.001	0,9998	>0,9999	>0,9999
	untreated vs. MOI 0.01	0,9999	>0,9999	>0,9999
	untreated vs. MOI 0.1	>0,9999	>0,9999	0,9999
	untreated vs. MOI 1	>0,9999	0,9139	0,0103
	untreated vs. MOI 10.0	0,0405	<0,0001	<0,0001
	MOI 0.001 vs. MOI 0.01	>0,9999	>0,9999	>0,9999
	MOI 0.001 vs. MOI 0.1	>0,9999	>0,9999	>0,9999
**MiaPaca2**	MOI 0.001 vs. MOI 1	0,9998	0,9487	0,0413
	MOI 0.001 vs. MOI 10.0	0,0466	<0,0001	<0,0001
	MOI 0.01 vs. MOI 0.1	>0,9999	>0,9999	>0,9999
	MOI 0.01 vs. MOI 1	>0,9999	0,9494	0,0582
	MOI 0.01 vs. MOI 10.0	0,1636	<0,0001	<0,0001
	MOI 0.1 vs. MOI 1	>0,9999	0,9089	0,0021
	MOI 0.1 vs. MOI 10.0	0,1664	<0,0001	<0,0001
	MOI 1 vs. MOI 10.0	0,0397	<0,0001	<0,0001

Table S3: Ranking of cancer cell lines from high to low susceptibility to oHSV1-FLT3L-mediated oncolysis

			OV FLTL3L MOI							
			0.001	0.01	0.1	1	10	AUC_mean_SUM	rank_MOI_sign	AUC_mean_SUM*rank-MOI_sign
AUC_mean	624-mel	melanoma	111,3631	97,32922	91,52297	57,02455	35,07661	392,3164032	4	1569,265613
	938-mel	melanoma	100,6257	97,94284	82,79856	43,547	31,59995	356,5140929	4	1426,056372
	LN229	glioblastoma	110,7998	105,3322	103,8794	89,28142	64,33509	473,6279133	5	2368,139567
	U87	glioblastoma	81,69452	78,26845	72,38763	54,40465	42,07353	328,8287736	3	986,4863207
	ASPC1	pancreatic cancer	74,55676	60,7918	45,31391	35,05238	32,68662	248,4014679	1	248,4014679
	BXPC3	pancreatic cancer	50,19023	41,38936	30,89918	24,29391	20,75899	167,5316794	1	167,5316794
	SUIT2	pancreatic cancer	110,2939	95,76675	92,34036	67,28308	49,09017	414,7742978	5	2073,871489
	Capan-1	pancreatic cancer	97,64269	86,07914	75,02972	59,18428	54,98381	372,919643	2	745,8392861
	SW1990	pancreatic cancer	95,84066	75,10869	52,83113	37,80406	30,68684	292,2713765	2	584,5427529
	PaTu8988t	pancreatic cancer	112,5555	89,96752	86,17049	55,0603	23,72818	367,4820018	4	1469,928007
	MiaPaca-2	pancreatic cancer	105,379	94,05018	98,28452	93,5314	78,20213	469,4472273	6	2816,683364

Table S4: Association between mutational profile of cell lines and their sensitivity to infection with oHSV1-FLT3L

	**BXPC3**	**ASPC1**	**SW1990**	**Capan-1**	**U87**	**PaTu8988t**	**624-mel**	**SUIT2**	**LN229**	**MiaPaca2**
BRAF	Val487_Pro492delinsAla						Val600Glu			
BRCA2				Ser1982Argfs*22						
CDK6					Pro148=					
CDKN2A	deletion	Leu78Hisfs*41<br>His93Profs*67						His83Tyr<br>Ala97Val	deletion	deletion
CDKN2C					deletion				deletion	
CTNNB1							Ser45Val			
EP300						Ex17-19del				
FLCN			Gln533Ter							
FZD10				Asn54Lys						
GLTT6D1				Trp198Ter						
GRM1				Tyr486Ter						
IDH1					Arg132His					
KRAS		Gly12Asp	Gly12Asp	Gly12Val		Gly12Val		Gly12Asp		Gly12Cys
LIFR									Pro1060Ala	
MAP2K4		deletion		Glu221Ter						
NF1					Phe1247Ilefs*18					
PTEN					c.209+1G>T					
RAD21									Gln132Ter	
SMAD4	deletion	Arg100Thr		Ser343Ter		deletion				
SMAP2				Ser255Ter						
TERT					c.228C>T				c.228C>T	
TP53	Tyr220Cys	Cys135Alafs*35	Pro191del	Ala159Val		Arg282Trp	Cys275Trp	Arg273His	Pro98Leu	Arg248Trp
AUC_SUM*MOI_rank	167,5316794	248,4014679	584,5428	745,8392861	986,4863207	1469,928	1569,2656	2073,871	2368,1396	2816,683

1

42ççà

Table S5: Composition of cell culture media per cancer cell line

Cell line	Medium (*Thermo Fisher Scientific*)	
**MELANOMA**		
**624-mel**	RPMI 1640	10% Fetal bovine serum (FBS) *(Greiner Bio-One)* + *10,000 units/mL* penicillin/streptavidin (pen/strep) *(Thermo Fisher Scientific)* + 2 µM L-glutamine *(Thermo Fisher Scientific)*
**938-mel**	RPMI 1640	10% FBS + *10,000 units/mL* pen/strep + 2 µM L-glutamine
**GLIOBLASTOMA**		
**U87**	MEM	10% FBS + *10,000 units/mL* pen/strep + 2 µM L-glutamine
**LN229**	DMEM	10% FBS + *10,000 units/mL* pen/strep + 2 µM L-glutamine
**PANCREATIC CANCER**		
**BXPC3**	RPMI 1640	10% FBS + *10,000 units/mL* pen/strep + 2 µM L-glutamine
**ASPC1**	RPMI 1640	10% FBS + *10,000 units/mL* pen/strep + 2 µM L-glutamine
**Capan-1**	IMDM	20% FBS + *10,000 units/mL* pen/strep + 2 µM L- glutamine
**MiaPaca2**	DMEM	10% FBS + 2,5% HI Horse serum (Thermo Fisher Scientific) + *10,000 units/mL* pen/strep + 2 µM L-glutamine
**SUIT2**	DMEM	5% FBS + 5% HI Horse serum + *10,000 units/mL* pen/strep + 2 µM L-glutamine
**PaTu8988t**	DMEM	10% FBS + *10,000 units/mL* pen/strep + 2 µM L-glutamine
**SW1990**	IMDM	20% FBS + *10,000 units/mL* pen/strep + 2 µM L-glutamine

Supplemental figures

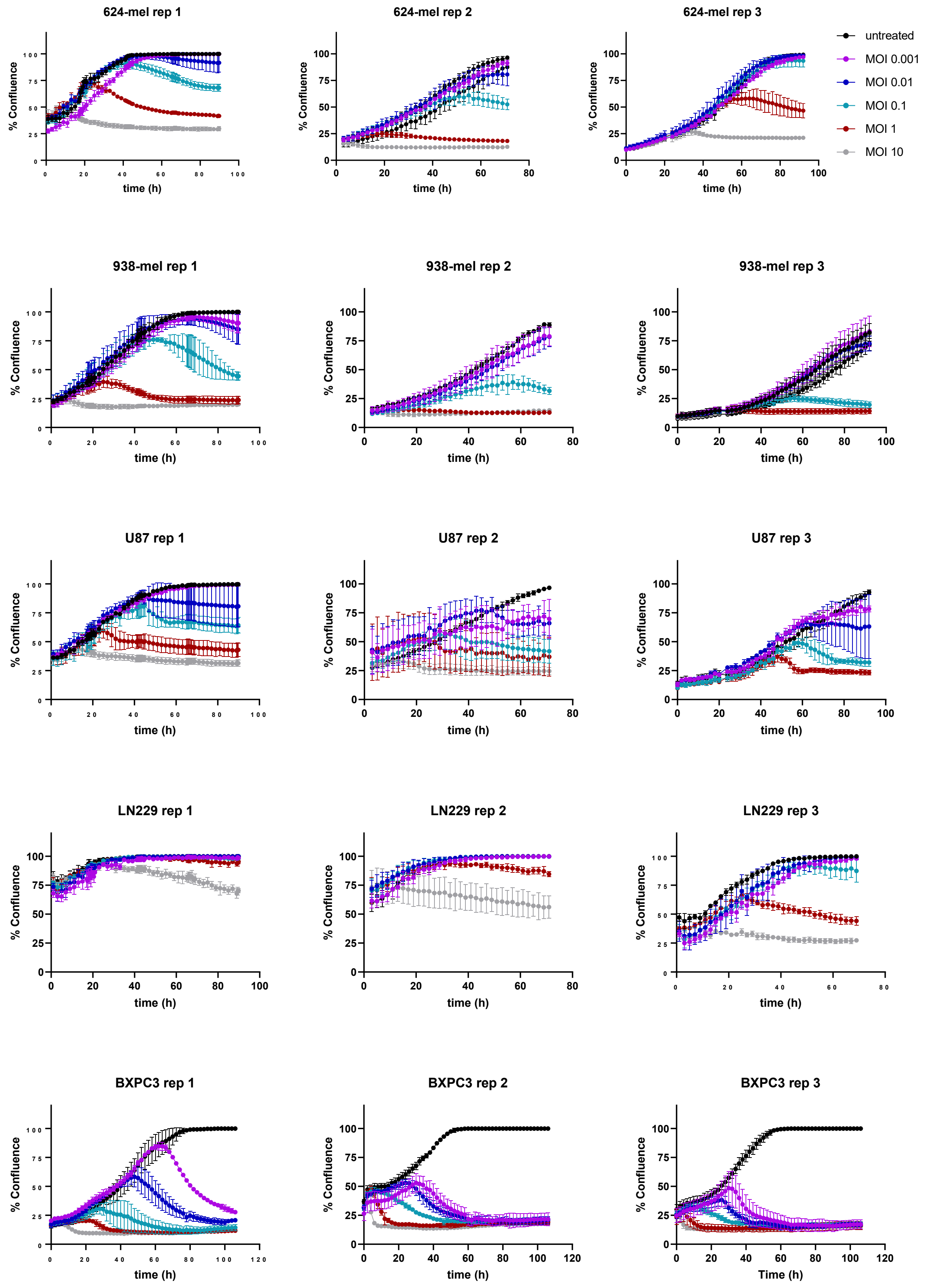

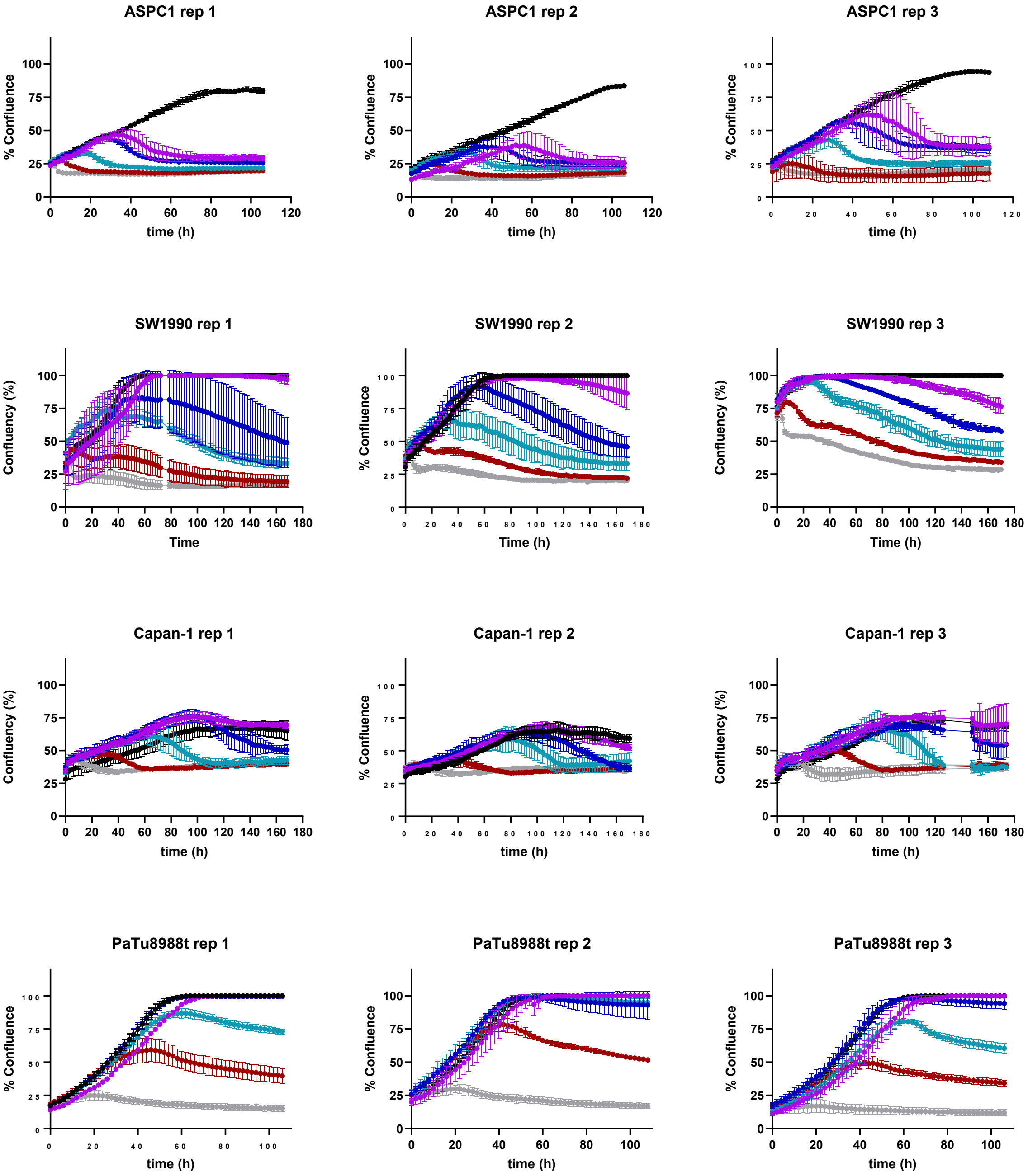
ASPC1 rep 1
% Confluence
time (h)
ASPC1 rep 2
% Confluence
time (h)
ASPC1 rep 3
% Confluence
time (h)
SW1990 rep 1
Confluency (%)
Time
SW1990 rep 2
% Confluence
Time (h)
SW1990 rep 3
Confluency (%)
Time (h)
Capan-1 rep 1
Confluency (%)
time (h)
Capan-1 rep 2
% Confluence
time (h)
Capan-1 rep 3
Confluency (%)
time (h)
PaTu8988t rep 1
% Confluence
time (h)
PaTu8988t rep 2
% Confluence
time (h)
PaTu8988t rep 3
% Confluence
time (h)

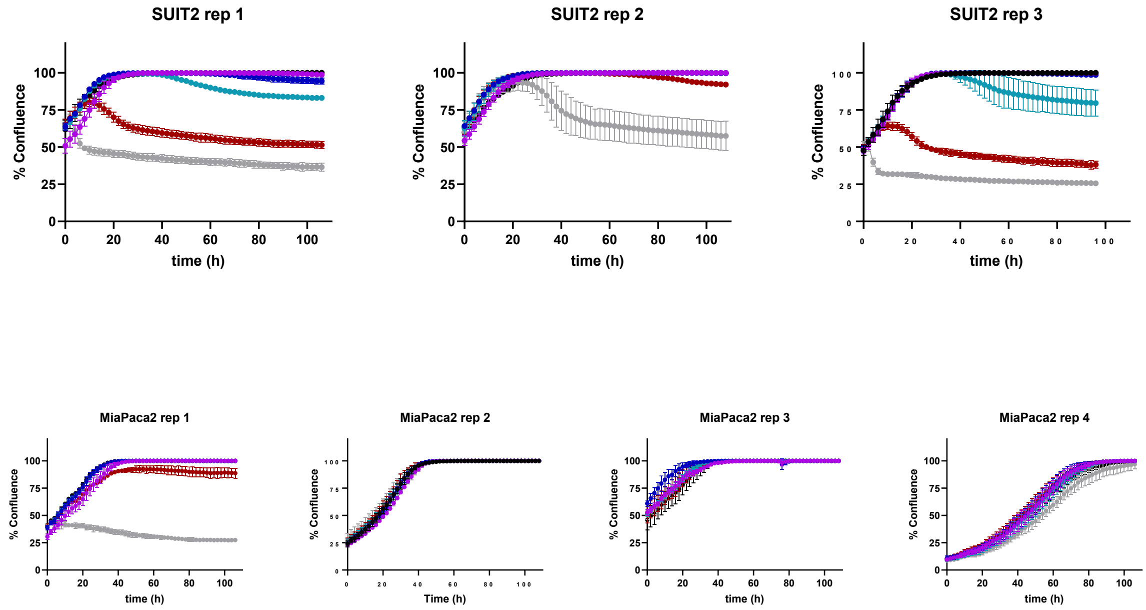

Figure S1. Inhibition of tumor cell growth by oHSV1-FLT3L. The growth of the different tumor cell lines was analysed in real time using Incuycte® live cell imaging. Cells were plated and left to adhere overnight, after which oHSV1-FLT3L was added at different MOIs ranging from MOI 0.001-10 (time 0). Percent confluence was calculated using the Incucyte® analysis software. Data points are depicted as a mean ± SD of 3 technical replicates. Each graph shows a biological repeat. The last experiment (3rd panel on the right) of Capan-1 has a gap between 127h-149h due to a technical issue.

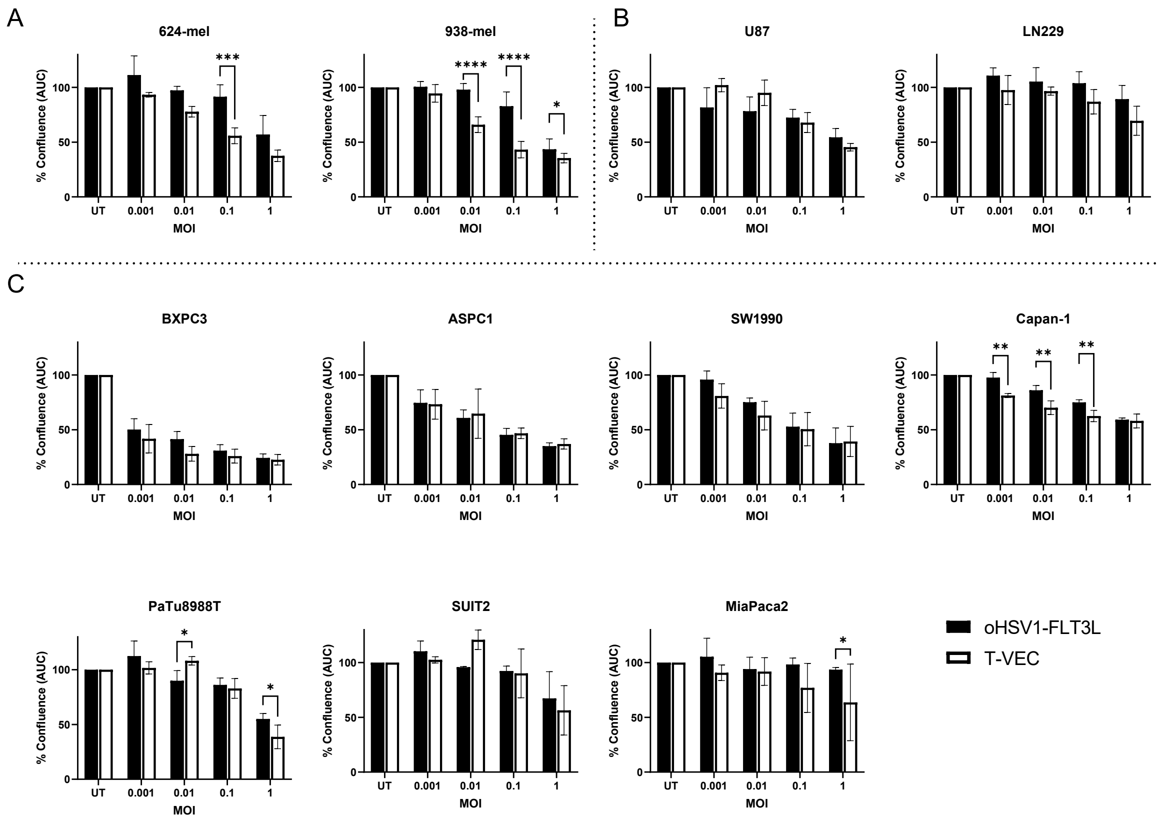

Figure S2: Comparison of cell growth inhibition induced by oHSV1-FLT3L to T-VEC. The percent confluence is displayed as the area under the curve (AUC) normalized to the untreated (UT) condition for **(A)** melanoma cell lines (624-mel, 938-mel), **(B)** glioblastoma cell lines (U87, LN229), **(C)** pancreatic ductal adenocarcinoma cell lines (BXPC3, ASPC1, SW1990, Capan-1, PaTu8988t, SUIT2, MiaPaca2). Bars depict the mean ± SD of 3 biological repeats with each individual data point depicting the mean of 3 technical repeats, except for MiaPaca2 with N=4. An ordinary two-way ANOVA with Sidák's multiple comparisons test was performed to compare oHSV1-FLT3L with T-VEC at each MOI. * $p \leq 0.05$, ** $p \leq 0.01$, *** $p \leq 0.001$, **** $p \leq 0.0001$.

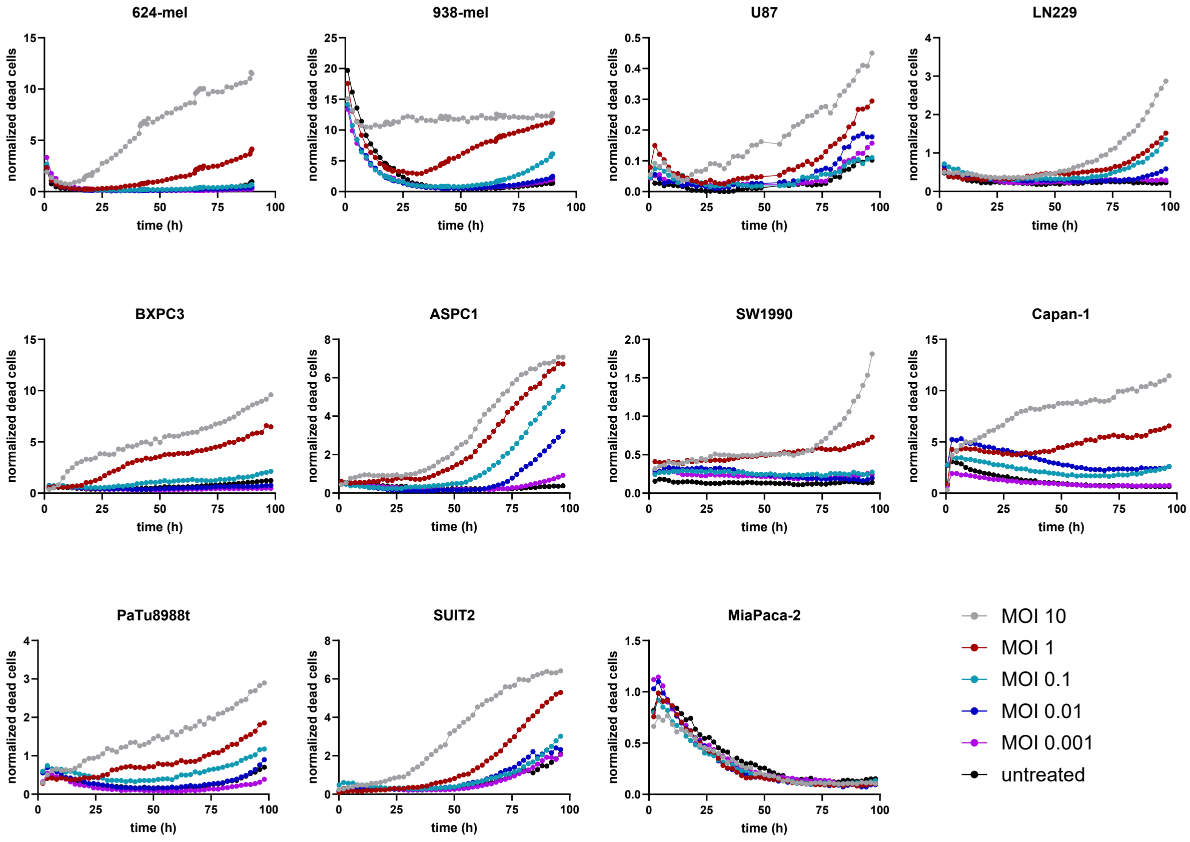

Figure S3: Induction of tumor cell death by oHSV1-FLT3L. Cell death induced by oHSV1-FLT3L in the different tumor cell lines was analysed in real time using Incuycte® live cell imaging. Cells were plated and left to adhere overnight, after which oHSV1-FLT3L was added at different MOIs ranging from MOI 0.001-10 in the presence of cytotox red dye (time 0). The number of dead cells per mm² was calculated using the Incucyte® analysis software and normalized to the percent confluence to obtain the normalized dead cell count. Data points are depicted as a mean of 3 technical replicates. Each graph shows a biological repeat.

A

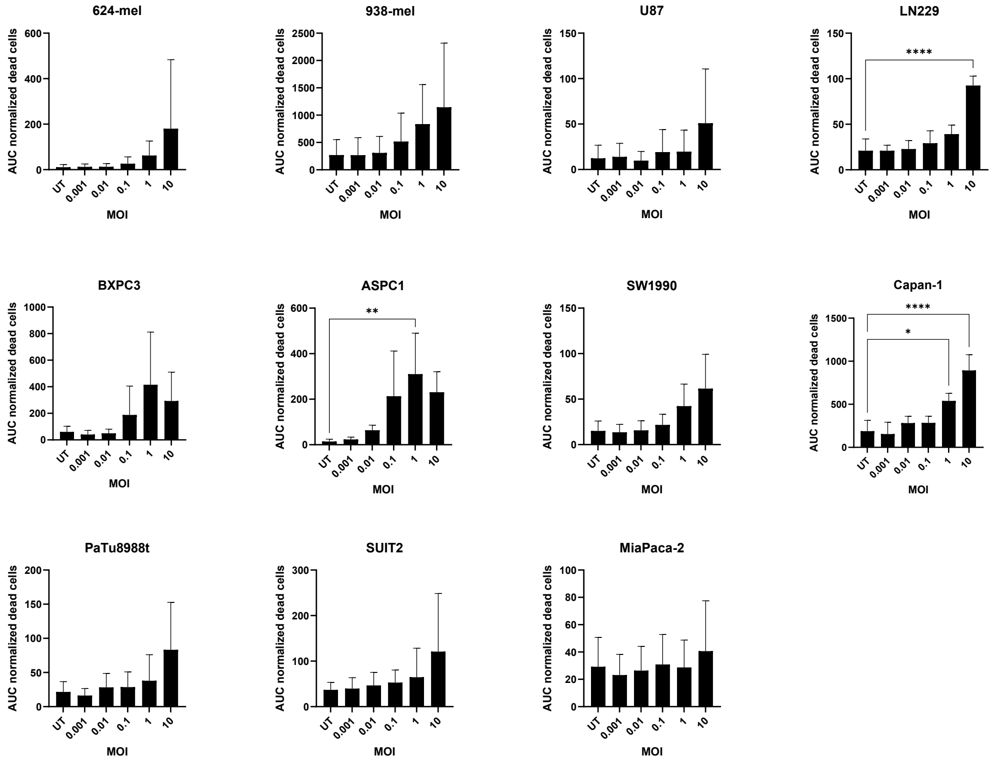
624-mel
938-mel
U87
LN229
BXPC3
ASPC1
SW1990
Capan-1
PaTu8988t
SUIT2
MiaPaca-2
AUC normalized dead cells
MOI
UT
0.001
0.01
0.1
1
10
****
**
*

B

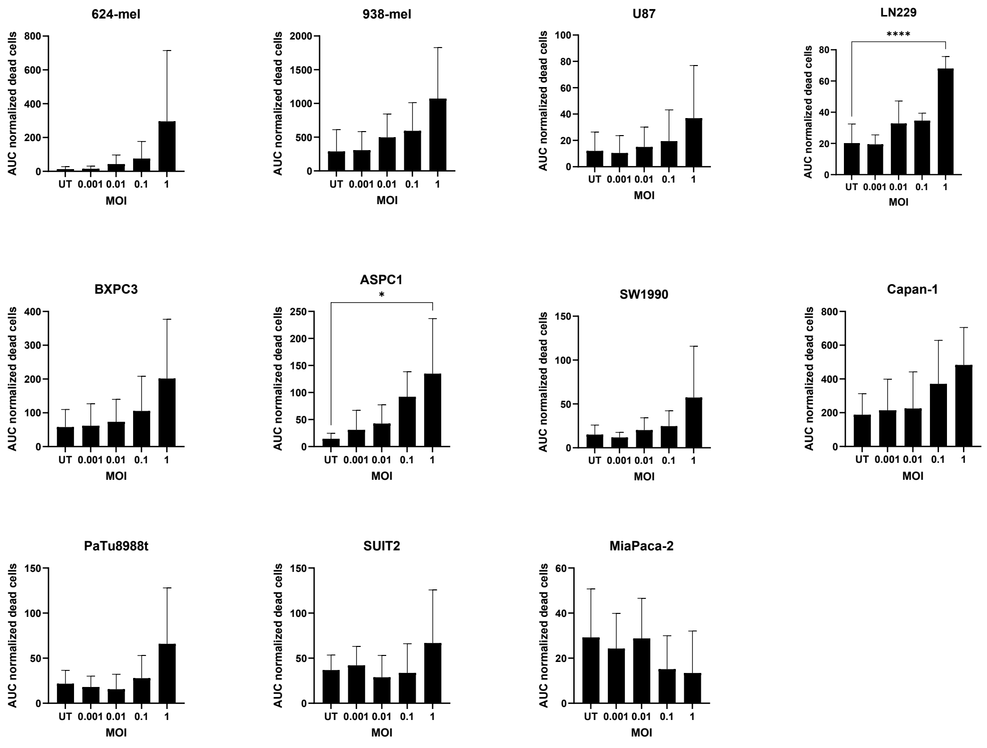
624-mel
938-mel
U87
LN229
BXPC3
ASPC1
SW1990
Capan-1
PaTu8988t
SUIT2
MiaPaca-2
AUC normalized dead cells
MOI
UT
0.001
0.01
0.1
1
****
*

Figure S4: Induction of cell death by oHSV1-FLT3L and T-VEC in different cancer cell lines. The number of dead cells per mm^2 was normalized to the percent confluence and the AUC is displayed for treatment with **(A)** oHSV1-FLT3L and **(B)** T-VEC. Bars depict the mean ± SD of 4 biological repeats, except for Capan-1, SW1990 and MiaPaca2 with n=3. An ordinary one-way ANOVA with Dunnett's multiple comparisons test was performed to compare the untreated (UT) condition with every MOI. * $p \leq 0.05$, *** $p \leq 0.001$, **** $p \leq 0.0001$.

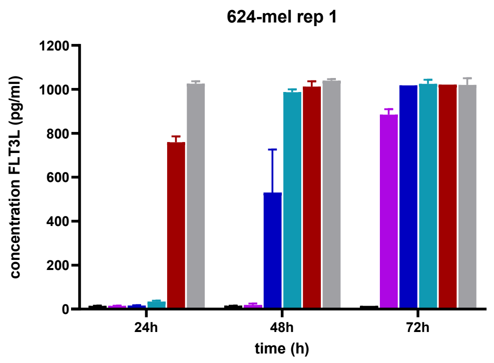
624-mel rep 1
concentration FLT3L (pg/ml)
0
200
400
600
800
1000
1200
24h
48h
72h
time (h)

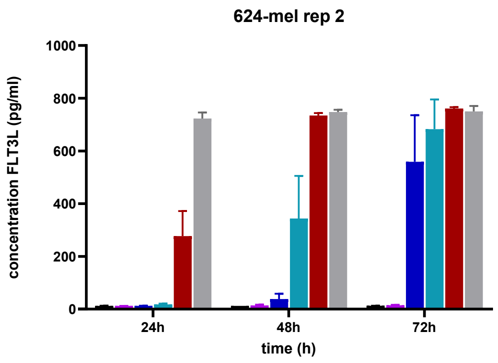
624-mel rep 2
concentration FLT3L (pg/ml)
0
200
400
600
800
1000
24h
48h
72h
time (h)

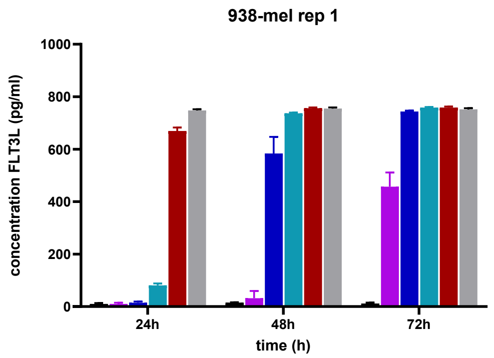
938-mel rep 1
concentration FLT3L (pg/ml)
0
200
400
600
800
1000
24h
48h
72h
time (h)

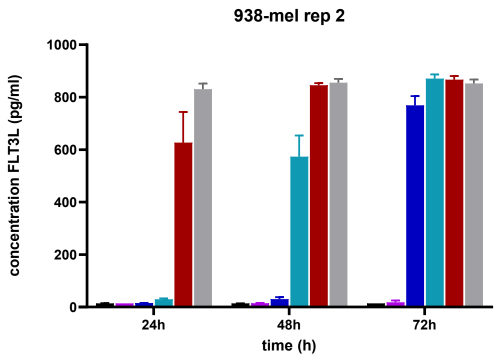
938-mel rep 2
concentration FLT3L (pg/ml)
0
200
400
600
800
1000
24h
48h
72h
time (h)

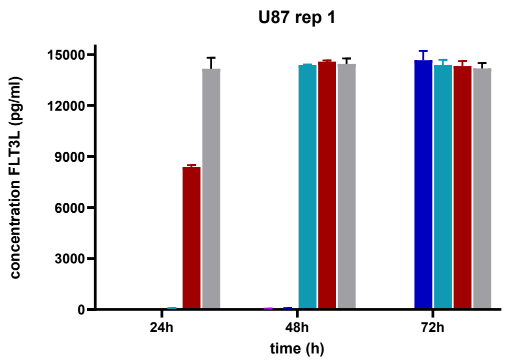
U87 rep 1
concentration FLT3L (pg/ml)
0
3000
6000
9000
12000
15000
24h
48h
72h
time (h)

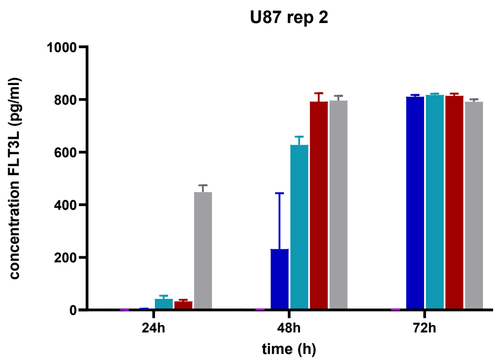
U87 rep 2
concentration FLT3L (pg/ml)
0
200
400
600
800
1000
24h
48h
72h
time (h)

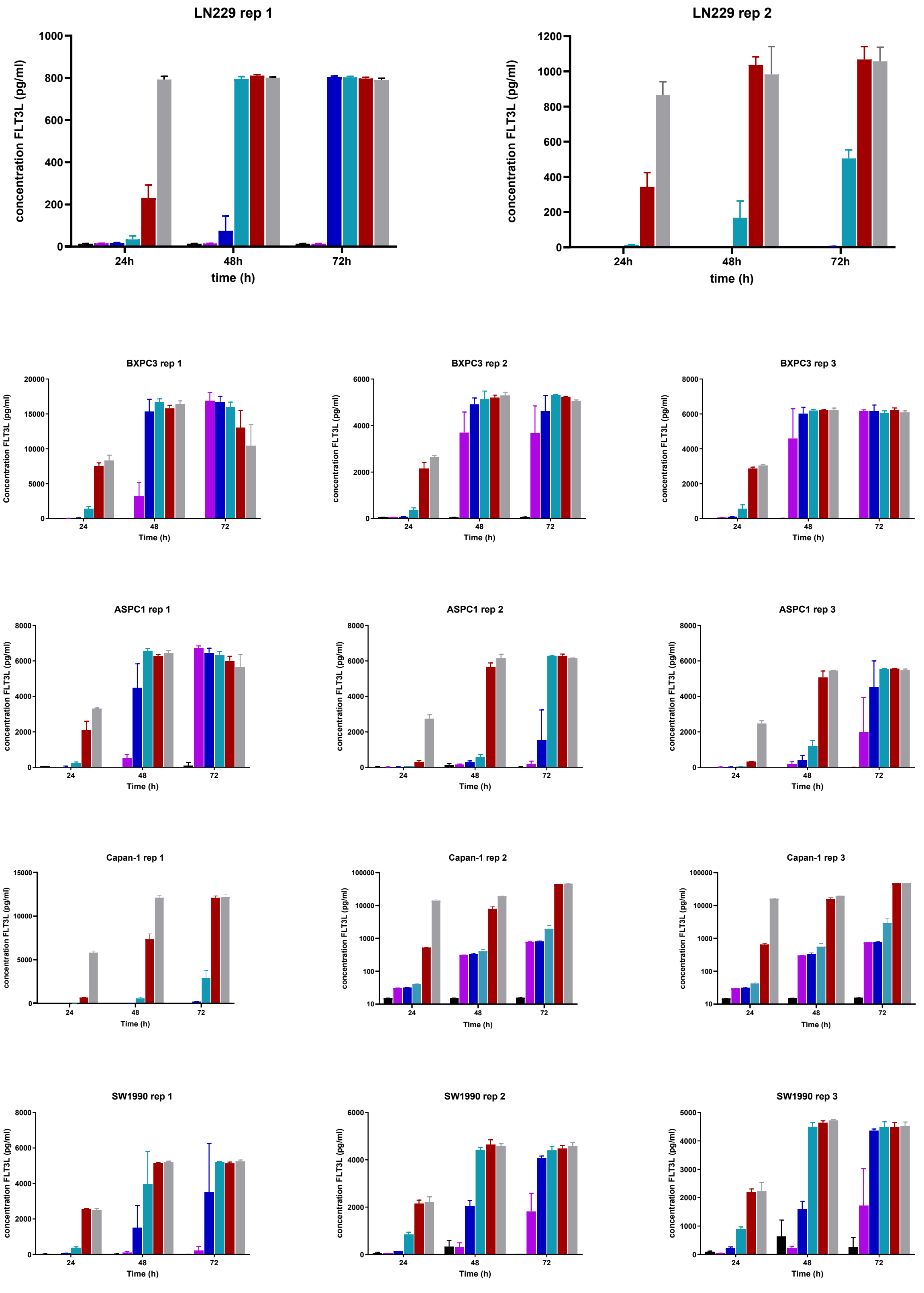
LN229 rep 1
concentration FLT3L (pg/ml)
24h
48h
72h
time (h)
LN229 rep 2
concentration FLT3L (pg/ml)
24h
48h
72h
time (h)
BXPC3 rep 1
Concentration FLT3L (pg/ml)
Time (h)
BXPC3 rep 2
concentration FLT3L (pg/ml)
Time (h)
BXPC3 rep 3
concentration FLT3L (pg/ml)
Time (h)
ASPC1 rep 1
concentration FLT3L (pg/ml)
Time (h)
ASPC1 rep 2
concentration FLT3L (pg/ml)
Time (h)
ASPC1 rep 3
concentration FLT3L (pg/ml)
Time (h)
Capan-1 rep 1
concentration FLT3L (pg/ml)
Time (h)
Capan-1 rep 2
concentration FLT3L (pg/ml)
Time (h)
Capan-1 rep 3
concentration FLT3L (pg/ml)
Time (h)
SW1990 rep 1
concentration FLT3L (pg/ml)
Time (h)
SW1990 rep 2
concentration FLT3L (pg/ml)
Time (h)
SW1990 rep 3
concentration FLT3L (pg/ml)
Time (h)

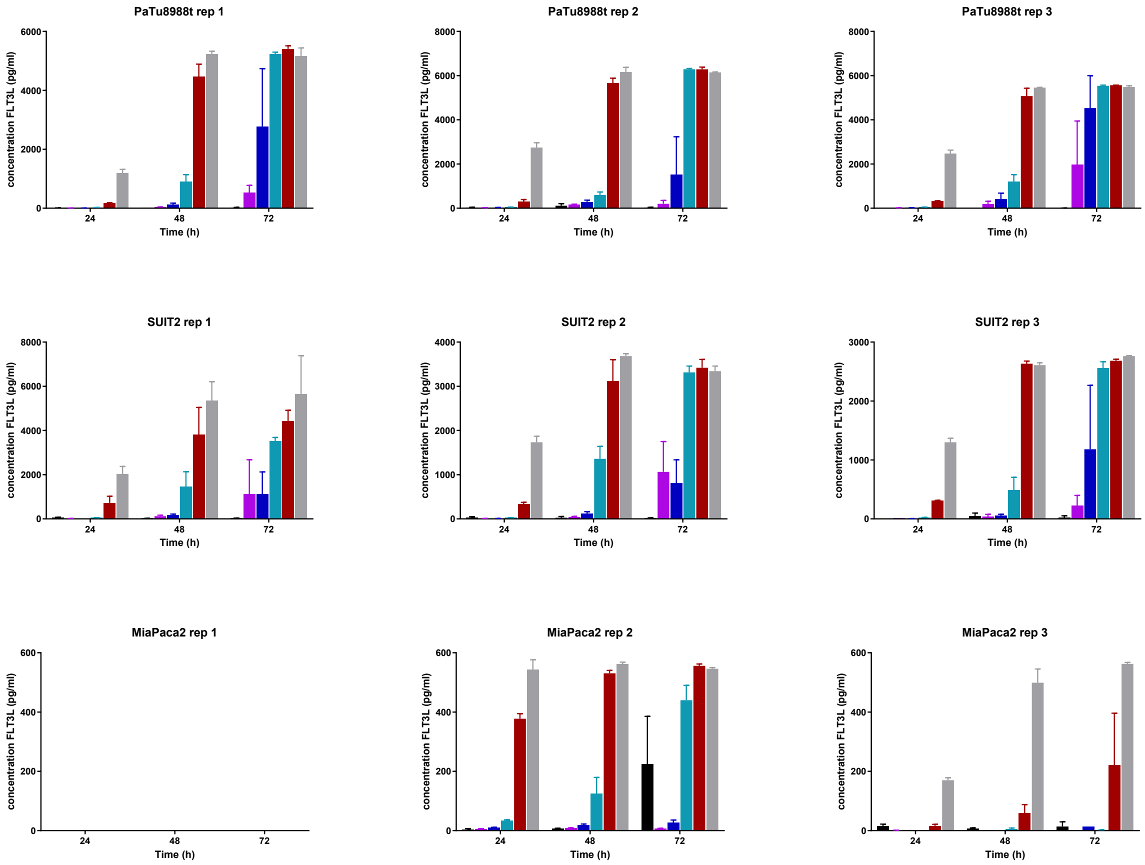

Figure S5: Replicate experiments of FLT3L secretion by different cancer cell lines upon oHSV1-FLT3L infection. Cancer cell lines were treated at different MOI (0.001 – 10) and supernatant was harvested after 24, 48 and 72h. FLT3L was detected in the supernatant of oHSV1-FLT3L-treated tumor cell lines by ELISA. Graphs depict individual biological replicate experiments with bars representing the mean ± SD of 3 technical replicates.

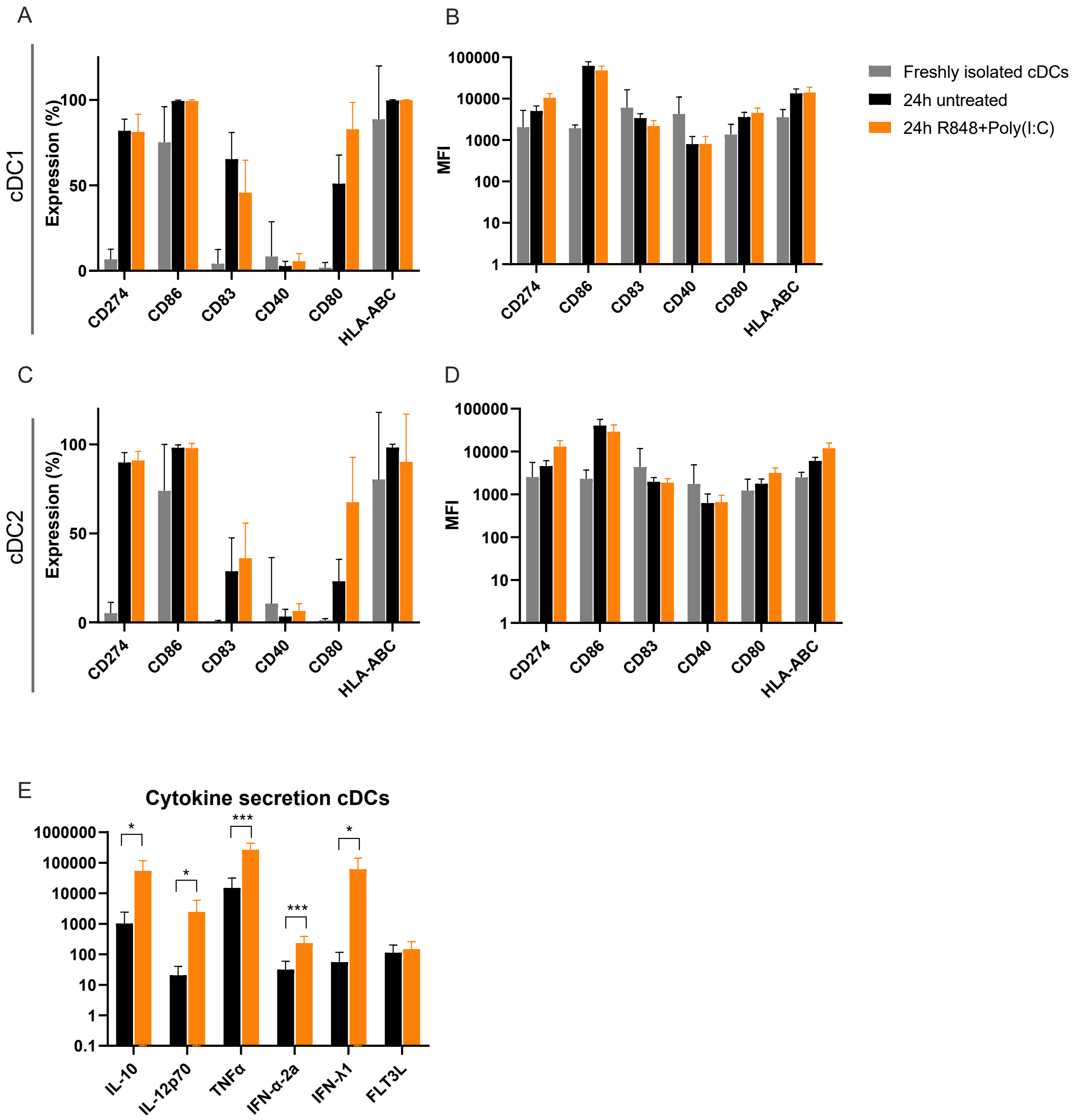

Figure S6: Effect of culturing BDCA-1⁺/BDCA-3⁺ cDC with or without R848/poly(I:C) on their phenotype and cytokine secretion. The phenotype of freshly isolated BDCA-1⁺/BDCA-3⁺ cDCs, 24h cultured BDCA-1⁺/BDCA-3⁺ cDCs (untreated), and BDCA-1⁺/BDCA-3⁺ cDCs cultured in the presence of R848 (1 mg/mL) + poly(I:C) (20 µg/mL) was assessed by analysis of expression of maturation markers (CD274, CD86, CD83, CD40, CD80, HLA-ABC) via flow cytometry after 24h incubation. (**A, C**) Percentage positive cells for every marker as well as (**B, D**) MFI of every marker is displayed for (**A, B**) BDCA-3⁺ cDC and (**C, D**) BDCA-1⁺ cDC subpopulation. (**E**) Cytokine secretion of the collected supernatant upon 24h culture of BDCA-1⁺/BDCA-3⁺ cDCs was analysed using MesoScale Diagnostics custom U-plex assays. All bars represent mean ± SD of 10 donors (10 separate experiments). Unpaired t-test was performed per cytokine. Adjusted p-values are displayed for all different comparisons and statistical significance was considered from $p \leq 0.05$ and * $p \leq 0.05$, ** $p \leq 0.01$, *** $p \leq 0.001$, **** $p \leq 0.0001$. MFI, mean fluorescence intensity.

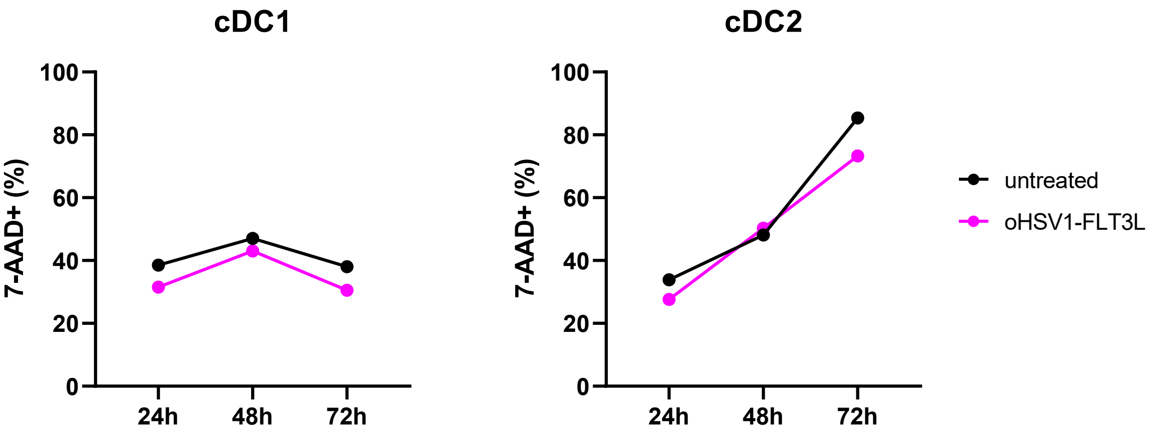

Figure S7: Effect of OV treatment on viability of BDCA-1⁺/BDCA-3⁺ cDCs. The effect of oHSV1-FLT3L on the viability of (**A**) BDCA-3⁺ cDC (cDC1) and (**B**) BDCA-1⁺ cDC (cDC2) over time. The percentage of cell death is indicated as the percentage of 7-AAD-positive cells after 24h, 48h, and 72h of culture (either untreated or oHSV1-FLT3L (MOI 1)). The graphs represent one representative experiment out of two. 7-AAD, 7-aminoactinomycin D.

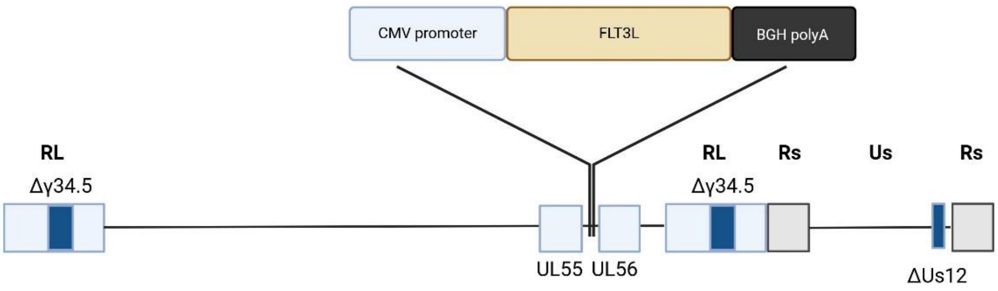

Figure S8: Graphical representation of the oHSV1-FLT3L viral vector

CMV: cytomegalovirus; BGH: bovine growth hormone; RL: repeat long; Rs: repeat short; Us: unique short

A

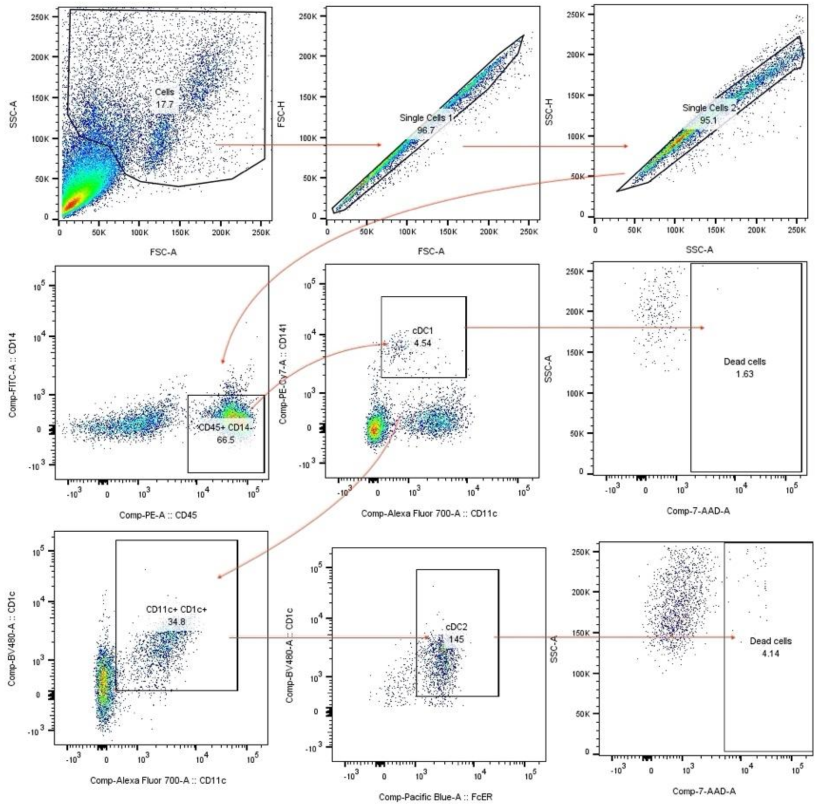

B

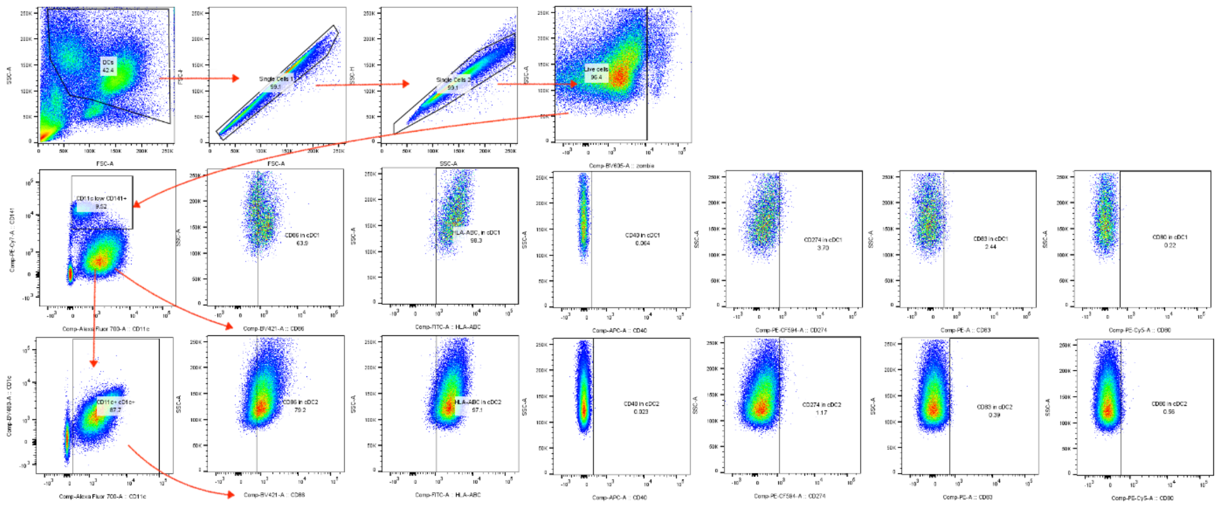

Figure S9: Gating strategy to assess cDC viability and maturation. We first gated cells based on FSC/SSC characteristics, followed by gating on single cells. **(A)** For viability assessment, CD45⁺ CD14⁻ cells were selected after which cDC1s were identified as CD141⁺ CD11c⁻/lo cells. A not gate was used to obtain all non-cDC1s and cDC2s were then gated as CD1c⁺ CD11c⁺ and CD1c⁺ FcγR⁺

population. On both cDC populations, dead cells were identified as 7-AAD^{+} cells. (B) To assess DC maturation, live cells were gated using Zombie yellow. Next, cDC1s were identified via gating CD141^{+} CD11c$^{-/lo}$ cells and a not gate was used to obtain all non-cDC1s and cDC2s were then selected as the CD1c^{+} CD11c^{+} population. On each cDC subtype, the expression of maturation markers CD86, HLA-ABC, CD40, CD274, CD83, and CD80 was assessed.
